# Rapid-Cycle Evaluation in an Early Intervention Program for Children With Developmental Disabilities in South India: Optimizing Service Providers' Quality of Work-Life, Family Program Engagement, and School Enrollment

**DOI:** 10.3389/fpubh.2020.567907

**Published:** 2020-11-30

**Authors:** Dinesh Krishna, Sankar Sahayraj Muthukaruppan, Aravind Bharathwaj, Ramasubramanian Ponnusamy, Bala Murugan Poomariappan, Sathiya Mariappan, Ayesha Beevi, Janna MacLachlan, Zoé Campbell, Chamila Anthonypillai, Marie Brien, Cathy Cameron, Marina Flatman, Lesley Perlman, Stephanie Seilman, Abhinayaa Jeyapragash, Lotte van der Haar, Joachim Krapels, Sankara Raman Srinivasan

**Affiliations:** ^1^Early Intervention Program, Amar Seva Sangam, Ayikudy, India; ^2^International Center for Disability and Rehabilitation, Rehabilitation Sciences Institute, University of Toronto, Toronto, ON, Canada; ^3^Handi-Care Intl., Toronto, ON, Canada; ^4^Utrecht School of Economics, Utrecht University, Utrecht, Netherlands; ^5^Porticus Global, Amsterdam, Netherlands

**Keywords:** rapid cycle evaluation, early childhood development (ECD), program engagement, school enrollment, community based rehabilitation (CBR), early intervention (EI), India, disability

## Abstract

**Background:** This paper explores how implementation and refinement of an early intervention (EI) program for children with delayed development was informed by an iterative, intentional and structured process of measurement. Providing access to early intervention therapy for children in rural areas of India is challenging due to a lack of rehabilitation therapists and programs. Following a biopsychosocial framework and principles of community-based rehabilitation, a non-governmental organization, Amar Seva Sangam (ASSA), overcame those barriers by designing a digital technology supported EI program in rural Tamil Nadu, India. Program objectives included providing service access; supporting program engagement, child development and school enrollment; and positioning the intervention for scale-up. This paper contributes to a growing body of literature on how program design and implementation can be informed through a cyclical process of data collection, analysis, reflection, and adaptation.

**Methods:** Through several strands of data collection, the design and implementation of the EI program was adapted and improved. This included qualitative data from focus groups and interviews with caregivers and service providers, and a mobile application that collected and monitored longitudinal quantitative data, including program engagement rates, developmental progression, caregiver outcomes, and school enrollment status.

**Results:** Measurements throughout the program informed decision-making by identifying facilitators and barriers to service providers' quality of work-life, family program engagement, and school enrollment. Consultation with key stakeholders, including caregivers and service providers, and data driven decision making led to continual program changes that improved service provider quality of work-life, program engagement and school enrollment. These changes included addressing gender-related work challenges for service providers; forming caregiver support networks; introducing psychological counseling for caregivers; providing medical consultations and assistive devices; creating community awareness programs; improving access to therapy services; focusing on caregiver education, motivation and support; and advocacy for accessibility in schools.

**Conclusion:** The process of using evidence-informed and stakeholder driven adaptations to the early intervention program, led to improved service provider quality of work-life, greater program engagement, improved school enrollment and positioned the intervention for scale-up, providing lessons that may be beneficial in other contexts.

## Introduction

In India, there are nearly 2.3 million children under the age of 6 with developmental disabilities, including 100,000 in the state of Tamil Nadu (1). Approximately 67,000 of these children live in rural and semi-urban areas and have no access to early childhood intervention services. Barriers include lack of rehabilitation centers in rural or semi-urban areas, long traveling times to urban centers, and high costs associated with private therapy. Barriers to establishment of more centers in rural and semi-urban areas include an inadequate number of rehabilitation specialists, high costs and lack of accessible transportation for families to bring their children to centers. Following a biopsychosocial framework and principles of community-based rehabilitation (CBR), a non-profit, non-governmental organization, Amar Seva Sangam (ASSA), designed, and implemented a community-based early intervention program called the mobile Village Based Rehabilitation–Early Intervention (mVBR-EI) program that provided access to early intervention (EI) service for children with delayed development in rural areas of Tamil Nadu, India, by leveraging mobile technology. The program was supported by the use of the mVBR-EI mobile application. Going forward, in this paper, the term mVBR-EI program will refer to the entire early intervention program and the term mVBR-EI app will refer to the mobile application that was used as part of this program to provide digital connectivity and case management.

The primary objective of ASSA's mVBR-EI program was to increase access to early identification of disabilities and provide EI therapy services to children identified with disabilities. The goal of EI services was to enhance children's physical, cognitive, communication, social and emotional development, reduce caregiver burden, empower families, and increase inclusion and participation of children with disabilities within their families, schools, and communities.

This paper describes the monitoring, evaluation, and learning systems employed by ASSA and how through several strands of data collection, the design and implementation of the mVRB-EI program was adapted, improved, and positioned for scaling. The paper seeks to contribute to the literature in various ways. First, research in early childhood development (ECD) has often focused on outcome measurement to evaluate the end point of an implementation process. Literature surrounding the dynamic process of change in ECD programs is less common, hence the call for improved implementation science in the 2018 special issue of Annals of the New York Academy of Sciences, “Implementation Research and Practice for Early Childhood Development” (2). Evaluation and measurement of both strengths and weaknesses is an important step in program implementation as it can better allow program managers to make decisions about whether to continue, change or stop a program (3). In the mVBR-EI program, we utilized a rapid cycle evaluation (4) to bring quasi-immediate change to our program.

Second, organizations in ECD are struggling to bring their programs to scale (5–7). A key condition for successful scaling is the idea of including stakeholders in decision-making throughout the design, implementation and evaluation of a program. To our knowledge, practical examples of how systematic consultation with stakeholders can be achieved and useful for all stakeholders are scarce in the ECD literature. Therefore, this paper provides two case studies from the mVBR-EI program to demonstrate how measurements can guide decision making and how rapid cycle evaluation and the inclusion of stakeholders in the process can help strengthen programs and advocate for successful scaling.

This paper first provides a detailed description of the mVBR-EI program to set out the context. This is followed by a brief overview of the methods, with further detailed description of methods as part of two in-depth cases that illustrate the rapid cycle evaluation model utilized in this program to generate evidence-informed change. These two cases are described within the context of a larger impact evaluation study of the program. The paper concludes with a discussion that reflects on the changes and results achieved in the program, combined with a general reflection on the utility of the rapid cycle evaluation model used.

## Program

### Program Setting

According to the World Health Organization's Early Childhood Development and Disability discussion paper, “If children with developmental delays or disabilities and their families are not provided with timely and appropriate early intervention, support and protection, their difficulties can become more severe—often leading to lifetime consequences, increased poverty, and profound exclusion” (8).

Policy makers in Tamil Nadu recognized that intervention at an early stage of development, in the pre-school years, could lead to better child development outcomes and greater school enrollment. The Tamil Nadu Government has established District Early Intervention Centers where children can access early intervention therapy including physiotherapy, occupational therapy, speech therapy, and special education provided by rehabilitation specialists to improve their development and function. These centers are in urban areas, often attached to Government Teaching Hospitals and therapy is provided free of cost to families. However, these centers are not accessible for the large percentage of children with disabilities who live in semi-urban and rural areas. The mVBR-EI program was implemented in eight rural locations (known locally as “blocks”) in the District of Tirunelveli, State of Tamil Nadu, in South India. A block encompasses a number of villages, each with a population of 70,000–100,000 people. This study occurred between April 2017 and August 2020.

### Program Design

#### Theoretical Framework

To address the objectives of our study, we utilized the World Health Organization's (WHO) International Classification of Functioning, Disability and Health (ICF) as a biopsychosocial framework, along with a community-based rehabilitation (CBR) strategy (9). The ICF is guided by the definition of disability as being the outcome of an interaction between a person's health condition and the context in which the person lives. CBR is a “strategy within general community development for the rehabilitation, poverty reduction, equalization of opportunities, and social inclusion of all people with disabilities” (10). In this respect, the ICF may be an excellent framework for implementing CBR programs with outcomes described at three levels—body, person, and society (11). The ICF provides the framework in assessing individuals, their communities, and environment to determine the factors that are creating the disability and provide structure for appropriate interventions. Using a “twin-track” approach, CBR promotes community-based inclusive development by working with people with disabilities, their community and society to provide services that address their needs and providing equal opportunities to enhance their capacities (12). CBR can be especially beneficial in providing low-cost services for those living in low-resource, capacity-constrained settings, which is often the setting for people living with a disability in rural areas of a country in the Global South (12, 13). A CBR approach is complex, multidimensional, multisectoral and bottom-up while providing a practical strategy for program implementation, monitoring, and evaluation (10, 13). Partnerships with relevant stakeholders are key, with emphasis placed on including the voices of people living with a disability and their families (10, 12, 13).

#### Service Providers and Training

The early intervention therapy service providers in this program consisted of Community Rehabilitation Workers (CRWs) and rehabilitation specialists employed by ASSA. CRWs were high school graduates and some had diplomas in community-based rehabilitation. All CRWs received an initial 10 days of training on how to provide early intervention services to children and support their caregivers in caring for their child. Ongoing training was provided during joint visits to children's homes with rehabilitation specialists, monthly case discussion meetings and 10-day enhancement training workshops that occurred every 6 months.

Rehabilitation specialists were physiotherapists, special educators and speech trainers who all had degrees or diplomas in their respective field of practice. In addition, the program had one occupational therapist and one speech therapist who provided consultative care. Rehabilitation specialists also received 10 days of initial training and enhancement training every 6 months in the clinical and operational aspects of providing early intervention therapy services.

All service providers were trained on how to provide family-centered care based on the ICF framework (9) and principles of CBR (10). Service providers were given resource materials, including impairment-specific, treatment-specific and operational manuals.

#### Screening and Assessment

In India, children are often identified as having a developmental delay when they attend an unrelated health-care appointment (14) or when they start school (8). To promote early identification of developmental delays in children, ASSA implemented screening programs. These were conducted by ASSA's CRWs in primary health centers along with government Village Health Nurses where children aged 0–3 years receive regular health check-ups and immunizations, and in Anganwadi Centers with government Anganwadi workers (pre-school teachers), where children aged 3–6 years attend pre-schools. To screen children in a standardized manner, the Trivandrum Developmental Screening Chart (TDSC) was used. The TDSC was developed and validated for use with children between 0–6 years old and administered by community-based workers in a similar setting to the mVBR-EI program, in rural South India (14). The tool is available in English and was translated into Tamil (the first language of most residents of Tamil Nadu), is easy to use and consists of 51 items assessing mental, motor and language skills, and showed high sensitivity and specificity when validated against the Denver Developmental Screening Test (14).

All children screened by ASSA had their TDSC data entered into the mVBR-EI app. If a child's screening showed potential developmental delays, a CRW working in the community where the child lived was notified on her mVBR-EI app to do a home visit for an initial assessment, which captured demographic data, medical history and developmental concerns provided by the child's caregiver(s). Rehabilitation specialists, including a speech trainer, special educator, and physiotherapist, would then visit the child's home to complete a comprehensive developmental assessment which included baselines assessments and validated, standardized measurement tools that are embedded in the mVBR-EI app. These tools included the Gross Motor Function Measure—GMFM-88, pediatric version of the Functional Independence Measure (WeeFIM), Functional Assessment Checklist for Programming (FACP) and the Communication Developmental Eclectic Approach to Language Learning (Com DEALL) Development Checklist.

Physiotherapists performed the GMFM-88 (15, 16), which measured gross motor development for children with cerebral palsy and the WeeFIM (17), which measured self-care, mobility, and cognition for all children. Special educators performed the FACP (18), which measured the child's performance in four domains: occupational, academic, social, and personal skills, and speech trainers performed the Com DEALL (19) which measured receptive and expressive communication and speech. These developmental tools were measured at baseline and repeated every 6 months. In addition, at their initial assessment, children with cerebral palsy were classified by a physiotherapist on their level of severity using the validated 5-level Gross Motor Function Classification System (GMFCS), with level I being the least severe and level 5 being the most severe (20) and this was entered into the mVBR-EI app.

From April 2017 to August 2019, a total of 52,036 children (26,717 boys, 25,319 girls) were screened as part of the mVBR-EI program and a total of 1,136 (2.2%) were identified as having delayed development. The primary developmental disabilities identified were cerebral palsy (40%), speech and communication disorder (26%), intellectual impairment without cerebral palsy (22%), orthopedic disabilities (8%), and autism (4%).

#### Family-Centered Services

Family-centered care (FCC) is considered best practice for planning and delivering pediatric health care services and is a component of the ICF model (9). Our study recognized the three core beliefs of FCC including respect for children and families, appreciation of the family's impact on the child's well-being, and family-professional collaboration (21). Therefore, in addition to child assessments, caregiver outcomes were measured in the mVBR-EI app by a rehabilitation specialist using the Tirunelveli Early Intervention Caregiver Assessment Tool developed by ASSA that consisted of existing scales and subscales, including the Family Empowerment Scale (FES) (22), Modified Caregiver Strain Index (MCSI) (23), and a locally developed scale to measure caregiver-child interaction. In addition, caregiver feedback on the program was collected through a series of questions rating their satisfaction with the EI therapy services they were receiving and open-ended questions on how to improve the program and an opportunity to share their challenges and successes.

FCC services endorse shared decision making and continuous, effective communication with families to ensure care is responsive to their needs and priorities (24). To facilitate family centered goal setting, caregivers identified child strengths, and needs through a tool called the Canadian Occupational Performance Measure (COPM) (25). The COPM has been shown to be a reliable, valid and responsive measure with families in a pediatric population (26). In addition, the mVBR-EI app tracked school enrollment and access to government services such as disability cards, grants, and assistive and adaptive equipment.

#### Therapy Planning

Based on these assessments, domains of delayed development were identified, and goals, barriers, and detailed intervention plans were developed by each rehabilitation specialist and entered into the mVBR-EI app. This plan was family centered by focusing on child strengths and areas of needs identified by family members. A CRW was assigned to each child and was responsible for implementing the tailored therapy plan with a focus on training caregivers to provide regular therapeutic activities to their child and integrate these activities into their child's daily life. Therapy visits also focused on caregiver education to foster positive attitudes, increase knowledge of their child's impairments and capabilities, and increase confidence in caring for their child. Caregivers were provided with resources and manuals in Tamil that covered impairment and intervention specific topics.

Rehabilitation specialists were available to support the CRW through monthly meetings, joint visits, phone calls, text messaging, or live video conferencing. Rehabilitation specialists reassessed the child every 6 months, including assessing the developmental and caregiver tools listed above and setting new goals and intervention plans in the mVBR-EI app. In this way, the majority of therapy was given by CRWs and caregivers, but supported by rehabilitation specialists through mobile technology. The program was delivered either within the child's home (home-based) or at a local Early Intervention Center (center-based). The therapy location (i.e., home or center-based) was determined in consultation with the child's family based on their preference. A number of factors might have influenced this choice, including distance from center, perceived and actual stigma in the community, transportation issues, and perceived severity of disability.

Therapy session lengths were determined based on the therapeutic needs of a particular child. For home-based care, each child was booked to receive 20 therapy sessions per 6 months for 30–90 min per visit from their CRW. In addition, joint visits by their CRW and treating rehabilitation specialist occurred once per month. In center-based care, children were booked to receive 60 therapy sessions per 6 months for 30–90 min per visit. Center-based therapy sessions alternated service provider, with CRWs and rehabilitation specialists guiding therapy and caregiver training on alternate visits. Therefore, the center-based program was more time and resource intensive (i.e., more therapy time and more time with rehabilitation specialists).

#### mVBR-EI App

The mVBR-EI app was initially designed in 2014 at ASSA with extensive input from a team of internal rehabilitation specialists and information technology (IT) experts. The app was then created by a team of third party IT consultants. The app was field tested between 2014 and 2017 with 212 children and their families and based on that experience, a major upgrade to improve functionality, user interface and user experience was completed prior to the launch of this study, which started in April 2017. The mVBR-EI's features are described above and include general assessment by CRWs, specialized assessments for rehabilitation specialists, goal setting using the COPM, individualized treatment planning using an extensive drop down menu and tracking of child access and participation, including school enrollment and government benefits. Community workers could see goals and treatment plans on their app and used it to follow through on regular therapeutic activities. Standardized developmental assessments described in section Screening and Assessment were all embedded in the app.

##### Dashboard, Scheduling, and Monitoring

The mVBR-EI app had scheduling and calendar features whereby the program's field team leaders planned and assigned schedules to all service providers based on activities required (e.g., screening camps, therapy visits, awareness programs, etc.). Scheduling for therapy visits were based on each child's needs, their location and availability. Service providers, including CRWs and specialists, had a calendar view whereby they saw what activities were scheduled for each work day and used GPS enabled check in and check out functions, so that travel and time spent providing therapy were monitored.

The dashboard feature of the mVBR-EI app allowed real-time monitoring for program management. If a child's development or caregiver assessment scores dropped, it created a flag in the system for program management and treating CRWs and specialists to review. The case was reviewed by the treating team, discussed with the management team, and if needed, discussed at monthly case rounds. Through these mechanisms, the intervention plan was re-adjusted as required. In addition, the dashboard allowed for a case management and data management system where various analyses could be performed at the level of the individual child, family or service provider and at the program level.

## Study Design and Methods

### Data Collection Methods

**Figure 1:**
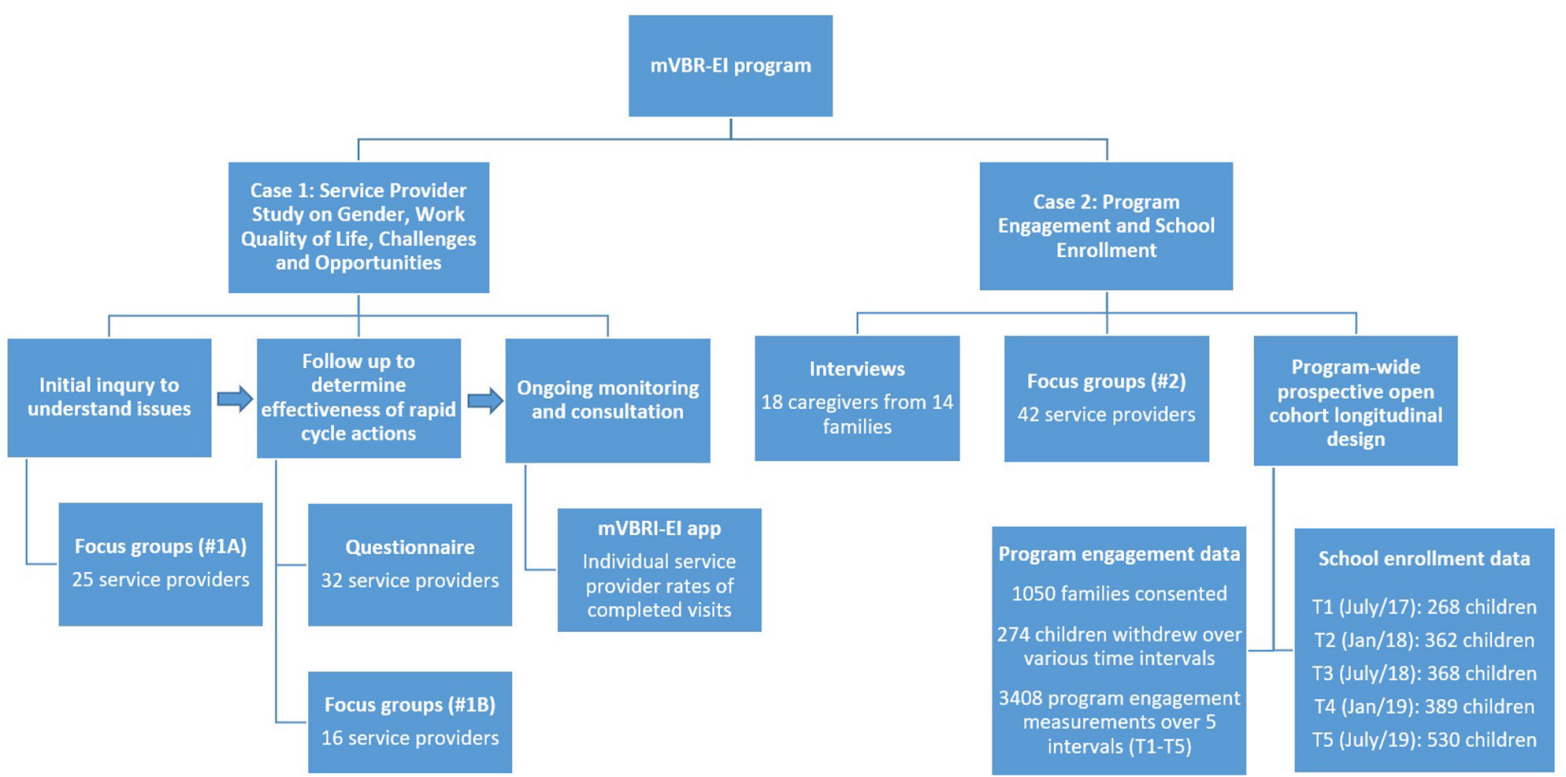
Summary of study methods and data sources.

In this paper, we employ two cases from the broader mVBR-EI program to investigate: What is the impact of employing a cyclical and iterative process of measurement and evaluation to support mVBR-EI program change? We sought to understand impact in terms of program outcomes, benefits, challenges and opportunities. To support this investigation, we drew on evaluation data from across the mVBR-EI program. The methods employed for each of the two cases are described in detail in the next section. The methods and data sources employed in both cases are summarized in Figure 1.

### Consent and Confidentiality

Written informed consent in accordance with the Declaration of Helsinki was obtained from service providers participating in the focus groups. Written consent was also provided by the primary caregiver of children enrolled in the program in consultation with the head of the family, caregivers and/or guardians of the child with development delay, as is customary in India's communal family system (27) for both the longitudinal cohort study and interviews. Consent documents were developed in English and translated into Tamil, and back translated to ensure integrity of translation.

Participation in the longitudinal cohort study and interviews had no influence on the care that the child received, and caregivers could choose not to participate in the study, but still have their child enrolled in the program. The researchers who conducted interviews and focus groups, collected data, and performed the data analysis were not involved in providing care to the family nor did they hold a supervisory role over the service providers. An independent translator with good working knowledge of Tamil and English assisted with translation and data collection. The translator accompanied the researcher to interview participants and signed a confidentiality agreement indicating that they understood and would maintain participant confidentiality.

### Ethics

Research ethics approval for this study was obtained from the Health Sciences Research Ethics Board, University of Toronto, Toronto, Canada (ref #34653) and the Research and Development Committee at Kalasalingam Academy of Research and Education, Madurai, India. In addition, the study was registered with an international clinical trial registry, ClinicalTrials.gov (NCT03202966).

### Rapid Cycle Evaluation

CBR programs are encouraged to use a management cycle to develop or strengthen their program (10). Given our methodology was guided by CBR principles, program changes to design and implementation were made in response to data collected and analyzed over the course of the program. These changes were the result of the application of rapid cycle evaluation. The goal of rapid cycle evaluation is to frequently evaluate a program after its implementation, which enables rapid identification of successes, needs, and opportunities (4). Changes can then be implemented more quickly rather than waiting to evaluate a program at its completion. This was important to the organization, as our goal was to advocate for further scale up of early intervention services in rural and semi-urban areas of the state to the Tamil Nadu state government. In order to be successful at this advocacy, it was essential that our program's outstanding challenges were addressed and mechanisms were in place to promote flexibility and adaptability. In the following sections, we describe the results obtained through measurement targeting three aspects of the program (service providers quality of work-life, family program engagement, and child school enrollment), through two cases that illustrate how results informed program changes through rapid cycle evaluation.

## Rapid Cycle Evaluation Cases

For each case presented below, the context is described followed by the data collection method utilized to inform changes. Results from the data collection and the changes in program design and implementation that followed are presented. Finally, we reflect on data that may reveal early effects of these implemented changes.

### Case 1—Service Provider Study on Gender, Work Quality of Life, Challenges, and Opportunities

#### Context

Traveling to rural and remote communities to provide home-based therapy can be challenging. It can positively and negatively impact service providers' health, economic status, family life, and sense of safety and well-being (28). In addition, the ability of service providers to attend a child's home for therapy visits plays an important role in program engagement rates, defined in our study as the proportion of planned therapy visits that were successfully completed. The majority (95%) of service providers in the mVBR-EI program are female, as it is more culturally acceptable and non-threatening for a woman to enter a household where the mother is usually the primary caregiver of a child with a disability. A study focused on female community health workers in India identified many factors that facilitated and impeded their well-being and ability to deliver home and community-based services, including sense of self-worth and motivation, community norms and beliefs as well as health system attitudes and practices (28). ASSA sought to examine the challenges and opportunities of female service providers working in our home-based program in order to make changes to improve their work-life experience. In addition, by examining barriers and facilitators to attending home visits, we could strengthen strategies to improve program engagement.

#### Methods: Focus Group 1A

A total of 10 female CRWs and 15 female rehabilitation specialists participated in 5 focus groups conducted to examine the relationship between gender and their work and its impact on their personal lives, relationships, health, sense of safety and well-being, social status, economic condition, and ability to perform work duties. Service providers were selected to participate on a voluntary basis and no incentive (financial or otherwise) was provided to participate in these groups. The groups consisted of 4–6 participants. One of the group facilitators was a newly hired staff member who spoke Tamil, had knowledge of the cultural context of the region, and did not know the focus group participants. The other group facilitator was a Canadian occupational therapist who helped develop this study, had a functional understanding of Tamil and knowledge of related literature.

There were two main sources used for developing the methods for gathering perspectives of service providers; a research article investigating female community health workers' experiences in navigating work challenges and addressing gaps in the healthcare system in India (28) and the Gender Equality Strategy Tool developed by Grand Challenges Canada (29). Components from these sources were included to gather information on potential limits to participation in work-related responsibilities, level of safety for women, gendered division of roles and link to decision-making power, and balance between work and life responsibilities. Focus groups were chosen with the intention to facilitate group discussion and enable the women to share their narratives and relevant experiences in a safe, comfortable, and judgement-free space.

Thematic analysis was used to interpret the data collected from the groups. First, the focus group recordings were translated and transcribed by group facilitators, with the removal of personal identification. When analyzing the focus groups, a six-stage thematic content analysis was conducted (30). The two researchers coded independently and read through the transcriptions and noted initial ideas (Phase 1), generated initial codes (Phase 2), searched for themes (Phase 3), reviewed themes (Phase 4), defined and named themes (Phase 5), and produced the report (Phase 6) (30). Program strategies informed by the results of these focus groups were then designed and implemented with an aim to improve service providers quality of work-life and ability to travel to rural communities to complete booked therapy visits.

#### Results

**Figure 2:**
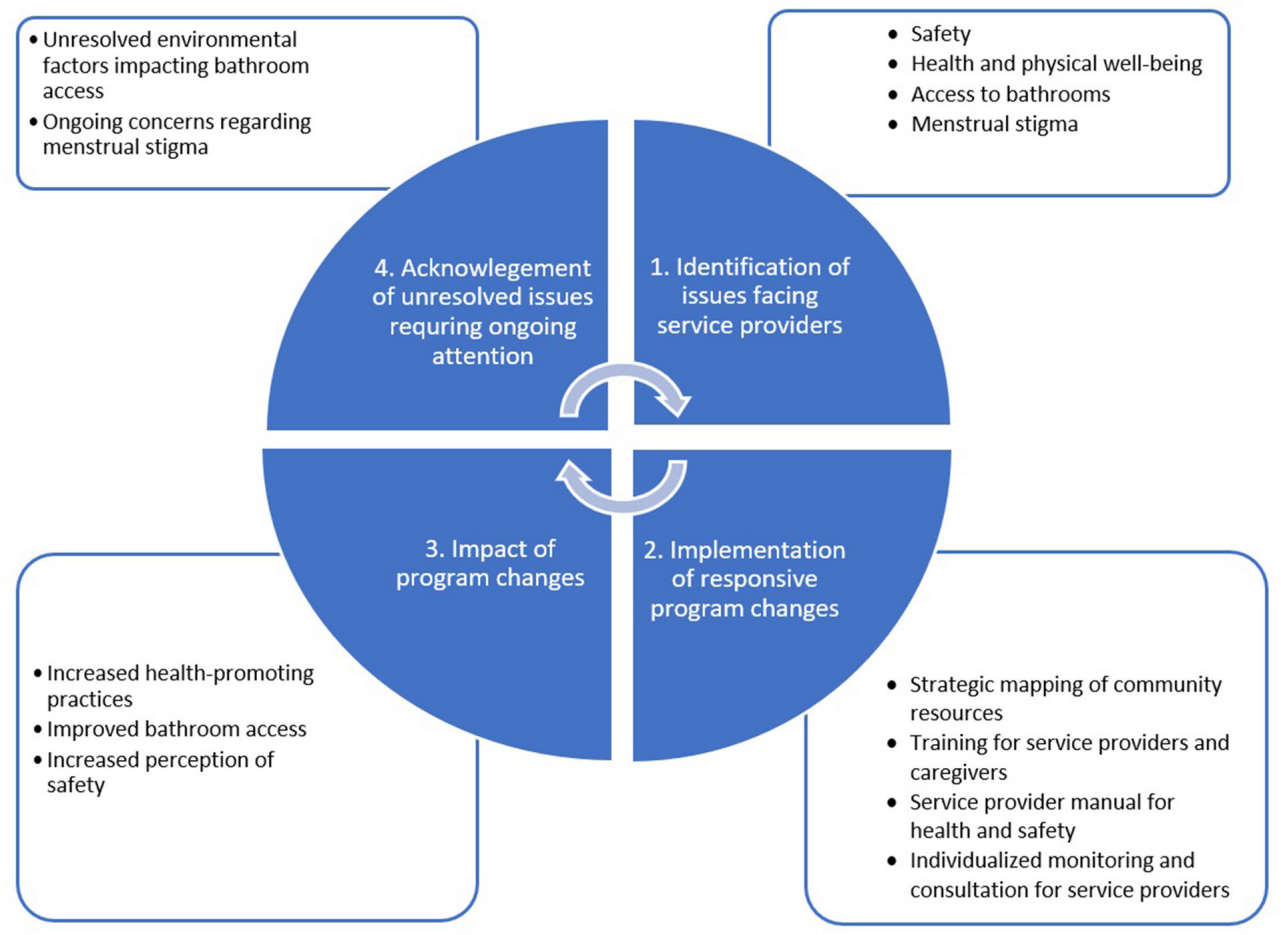
Summarized findings from Case 1, including the issues identified, rapid cycle evaluation actions taken and indications of effectiveness.

Figure 2 summarizes findings from this case, including the issues identified, rapid cycle evaluation actions taken and indications of effectiveness. Service providers in focus groups reported that working in the early intervention program at ASSA contributed to improving their self-confidence, economic status, and relations within their own family and community members. The focus groups gave space for the service providers to share the challenges related to their work with specific examples of encounters, their reactions and impact on their well-being and their ability to attend home visits and perform their work duties. The common issues raised can be broadly summarized under safety, health/physical well-being and access to bathrooms.

Many service providers reported at least one instance that they felt threatened, uncomfortable, or isolated when traveling via walking or public transit in the villages to conduct home visits as part of their work. It was common for the women to be approached particularly by single or groups of men, asking them why they were “roaming” around the community. Sometimes, the women reported being followed for several blocks and other times being asked to never visit the place again. They felt that they were unwelcome and judged negatively in these situations, stating that it was uncommon for a woman to be walking alone.

The participants mentioned numerous issues related to the nature of work and their physical well-being. They identified the long hours spent walking in the field and exposure to extreme heat contributing to various skin conditions, fatigue, and body pain. Gender specific issues were brought up by many of the women in terms of a lack of accessibility to toilets in their regular workday, especially when menstruating. The women shared that they often had to go without using a bathroom over the entire work day because many homes they worked at did not have a bathroom, locations of safe public bathrooms were not known to them, or they felt shy to ask caregivers to use their bathroom. This often led the women to abstain from drinking water and other fluids during the workday to minimize chances of needing to use a bathroom, which led to dehydration. This lack of accessibility to bathrooms was an amplified issue when the workers were menstruating, as there were no facilities to change their sanitary napkins. The women also described a stigma related to a woman being impure or “dirty” when they were menstruating, which dissuaded them from asking to use families' bathrooms. In addition, there were explicit requests from caregivers that they not enter their house during menstruation. Due to lack of accessibility to bathrooms and stigma, menstruation became a reason for missed therapy visits and being absent from work. In addition, concerns about dehydration, especially during menstruation, led to missed workdays.

#### Rapid Cycle Evaluation Action Taken

In light of the above results, ASSA developed strategies for service providers to increase the workers' work-related sense of safety, healthy practices, use of and accessibility to bathrooms, and reduce menstrual stigma. The strategies, described below, aimed to improve service providers' work-life well-being and ability to complete planned home visits.

##### Strategic Resource Mapping of Community Resources for Service Providers

ASSA management mapped out which families being served in the program had bathrooms. Therapy schedules were created so that workers had access to bathrooms in at least one home per half-day. Service providers were provided with a list of safe bathroom and rest spaces in every village including service users' homes and safe community spaces such as community centers or village leaders' homes. ASSA contacted village leaders in every village and explained the work our service providers were doing and gained their explicit support. Contact information for these village leaders and ASSA management were provided to service providers so they could make contact in case of a safety concern.

##### Service Provider and Caregiver Education

During their semi-annual 10-day training, all service providers were educated on measures they could take to stay safe while traveling in rural communities, including general safety measures such as traveling in groups and specific actions to be taken when feeling unsafe, such as calling village leaders. In addition, training on the importance of, and solutions to, maintaining healthy habits like using the bathroom more frequently during work, drinking fluids, taking care of themselves during menstruation and maintaining menstrual hygiene were given. They were educated about myths and stigma related to menstruation and encouraged to develop confidence to ask caregivers to use their bathroom during home visits and to enter houses during menstruation. A manual was also created to educate and provide tips to service providers regarding menstruation and health (particularly hydration, hand-washing, and menstrual hygiene). Safety concerns were addressed through the manual, with tips for safety and who to contact when concerned about safety.

Caregivers were educated about service providers' concerns around bathroom accessibility and menstruation-related stigma during a parent consultation meeting in which 604 caregivers of 312 children in the program attended. A simple hand-out was distributed to all caregivers in the program, including those who did not attend the meeting. It highlighted the importance of allowing service providers bathroom access and addressing menstrual stigma, including education around safety and acceptance of women working in their homes during menstruation. Awareness programs were created and provided to people living in the communities we worked in, in order to educate them on child development, child disability and early intervention therapy. By doing this, the community could gain greater understanding of the services ASSA was providing, thus reducing behaviors that threatened safety, such as questioning the presence of our service providers in the villages.

##### Monitoring and Consultation for Service Providers

Through the mVBR-EI dashboard, the management team was able to generate reports on individual service provider's rates of completed visits. Those with low rates had meetings with management to discuss individual challenges and barriers. Based on these consultations, individual solutions were instituted. For example, one service provider informed management that the route to clients' homes had frequent public transportation cancellations. Community leaders in her working area were informed and they raised funds to purchase a motorcycle for her to use for reaching children's homes. Another example was junior service providers being provided with peer mentorship from more senior service providers in order to improve their job satisfaction and ability to provide service.

#### Indications of Effectiveness

##### Methods: Questionnaire and Focus Group 1B—Effectiveness of Strategies to Address Gender-Related Work Quality of Life and Work Challenges

In order to examine the effectiveness of strategies implemented to address challenges described by service providers, a post-implementation study was conducted. The study consisted of a questionnaire and three focus groups, which focused on health, bathroom use, menstruation, and safety, after the implementation of the rapid cycle action. Convenience sampling was used to recruit participants including some who had participated in focus groups 1A and some who had not. There was a total of 32 service providers who completed the questionnaire and of those, 16 participated in one of three focus groups.

Six overarching themes for both the questionnaire and focus groups were created to address the impact of training, manual and resource mapping on health, safety, menstrual stigma, and bathroom use and access. The questionnaire used Likert-scale self-administered questions in paper format that required participants to rate the impact of the rapid cycle evaluation action from 1 to 5, where 1 indicated no positive impact and 5 indicated very strong positive impact. Focus groups were then conducted with participants to understand collective and individual perspectives. Descriptive statistical techniques, including means and frequency distributions, were used to summarize the quantitative information obtained from the questionnaires. Focus groups were recorded and transcribed verbatim by two researchers. When analyzing the focus groups, the same six step thematic content analysis that was performed for focus group 1A was conducted.

##### Results: Questionnaire and Focus Group 1B

**Figure 3:**
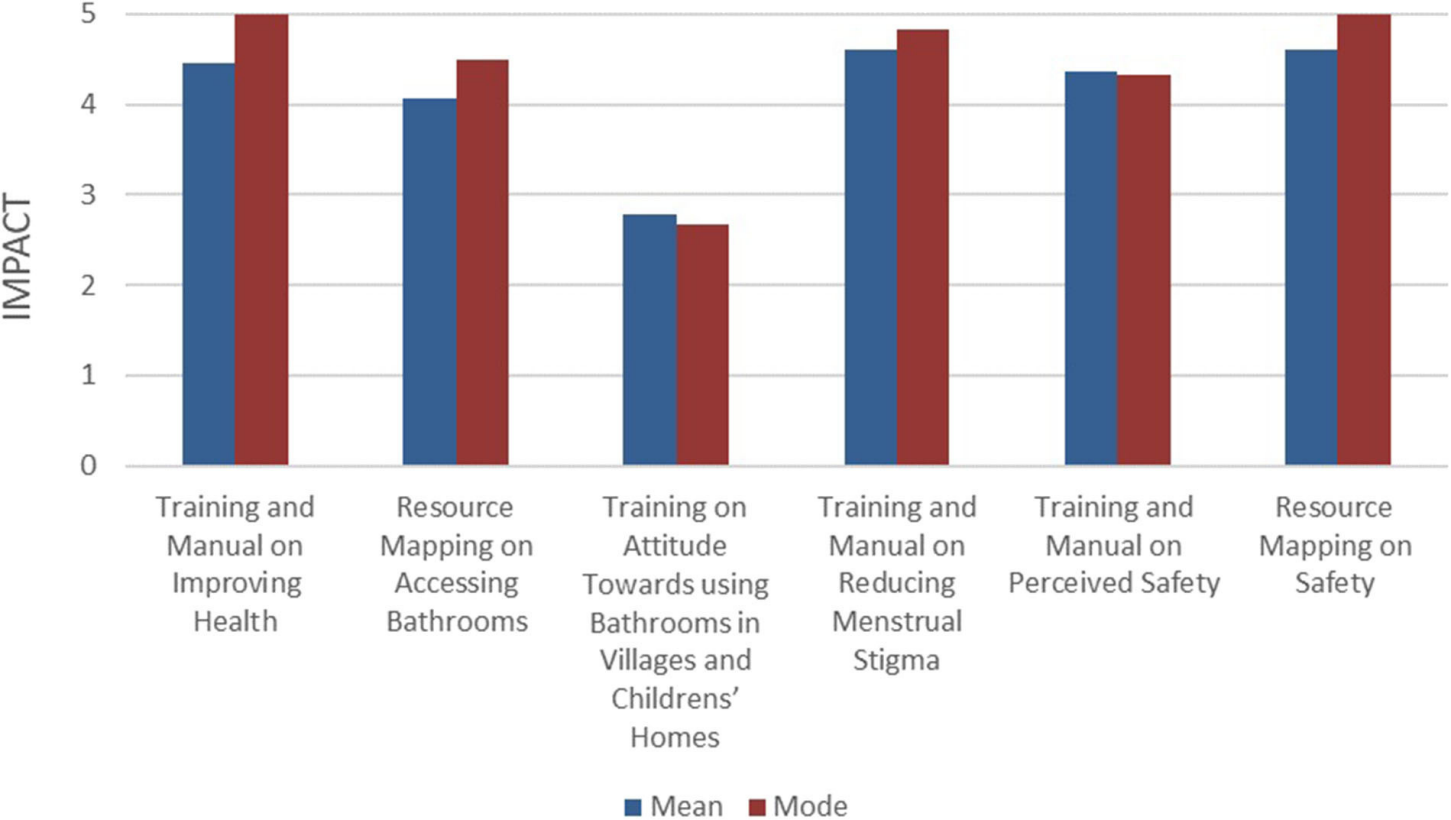
Impact of rapid cycle action addressing service provider challenges-questionnaire results.

Figure 3 highlights the results of the questionnaire. Overall, the results demonstrated that the training, manual and resource mapping had a major impact on improving service providers' health, access to bathrooms, their attitudes toward menstruation during work and their perceived safety. The impact on their attitude toward using bathrooms in the service users' home and in community spaces was moderate. The results of the focus groups mirrored the questionnaire, with feedback indicating an overall positive effect of training and manual on improving healthy practices including hydration, using bathrooms and menstrual hygiene practice; and resource mapping on improving bathroom access.

The focus groups additionally provided more nuanced insights. There were some concerns that lingered from initial focus group (1A) results, such as environmental factors (e.g., harsh weather conditions, poor hygiene in family or community bathrooms), which negatively impacted their physical well-being and continued to prevent use of bathrooms. While there was indication in focus group 1A that workers felt shy to ask caregivers for use of their bathroom, focus group 1B illustrated that interpersonal factors such as rapport and trust were important to create space for open communication and decreased fear of judgment for service providers. There were continued concerns expressed among participants related to attending work during menstruation, including discomfort with defying socially and culturally accepted practices for women and undervaluing the family's wishes for women not to enter the home during menstruation. As far as safety concerns, the participants stated that the connection with village leaders, their contact information and the awareness programs had a positive impact on reducing threatening behaviors and improving perception of safety while working in rural communities.

### Case 2—Program Engagement and School Enrollment

#### Context

An essential component to providing early intervention therapy services is to gauge the level of demand and acceptance by service users. ASSA recognized that not all families may share the same level of interest or ability to engage in EI services. Factors such as home stressors, personal priorities, work obligations, competing childcare duties, transportation, and stigma may impact a family's interest or ability to participate in EI services. As ASSA values equity in service accessibility, we sought to better understand the barriers and facilitators for program engagement for families, so that barriers could be addressed, enabling greater engagement by more families.

Overall, 72% of 5-year olds with disabilities in India have never attended any educational institution (31). The state of Tamil Nadu has been one of the most successful states in India at implementing programs under the Education for All—Integrated Education for Disability program, which aims to improve school enrollment and integration for children with disabilities through the implementation of individualized education programs, use of resource teachers and resource classrooms, provision of therapy and aids in school, transportation support, and home school support (32). However, despite these measures, school enrollment for children with disabilities in Tamil Nadu remains low with 23% of all children with disabilities aged 5–19 having never attended an educational institution, while 99.4% of all children attend school (31). Given this disparity, ASSA made it a priority to address school enrollment at an early age as part of the mVBR-EI program.

Since program engagement and school enrollment are interconnected and have many shared aspects related to caregiver challenges, it was felt that these two aspects should be studied and reported together.

#### Methods

Qualitative and quantitative data were employed to inform the Rapid Cycle Evaluation in this case (see Figure 1). The main purpose of the focus groups and interviews was to generate information that could inform program changes, while the main purpose of the longitudinal cohort study was to observe potential effects of the changes on the outcomes sought by the program.

##### Focus Group 2 and Interviews—Child Program Engagement and School Enrollment

Participants for the qualitative data informing this case were selected through purposeful sampling of families with children receiving care in the mVBR-EI program by independent researchers not involved in service provision to children. Families were chosen to represent a broad cross-section of participants based on a variety of criteria: program engagement (low, medium, high), gender of child (female/male), age (0–3/4–6), school enrollment status (yes/no), diagnosis, and location of therapy (home/center). In total, 30 families were selected and 14 agreed and were able to participate.

Most interviews were conducted with mothers (*N* = 8), although fathers (*N* = 3) and a combination of mother and father (*N* = 3) were also interviewed as primary caregivers. A convenience sample of service providers (rehabilitation specialists and CRWs) were invited to participate in focus groups. Eight focus groups were held with a total of 32 rehabilitation specialists and 10 CRWs participating. No incentive (financial or otherwise) was provided to caregivers or service providers.

The interviews and focus groups were held in locations that were considered convenient and private by the participants (e.g., home, ASSA center, sitting outside, etc.). Data collection occurred in Tamil, with English translation by an interpreter. Data were analyzed through coding of emergent categories by the primary investigator. An iterative, constant comparison approach was used whereby analysis began immediately following each focus group and interview, and informed future data collection. Focus groups and interviews were audio-recorded and summary notes taken. In addition, field notes regarding any observational or contextual details not captured on the audiotape were also collected to supplement the analysis.

##### Longitudinal Cohort Study

A prospective open cohort longitudinal design was used to evaluate the effectiveness of the mVBR-EI program. The primary objective of this prospective design was to assess change over time of child and family outcomes, including all the outcomes listed under the program design section above (Program Design). A primary caregiver was identified for each child enrolled in the mVBR-EI program. A primary caregiver was defined as the person who was most involved in the day-to-day care of the child. Caregivers were included if they were 18 years of age or older and were able to understand Tamil. Children were included if they were enrolled in the mVBR-EI program and their primary caregiver consented for the child's data to be included in the study.

*Program engagement: definition and calculation*. Program engagement was defined in our study as the number of therapy visits completed divided by the number of therapy visits planned for a child. Completed therapy visits were electronically monitored through the GPS functionality of the mVBR-EI app. Every time a CRW and/or rehabilitation specialist visited a child's home, they used the check-in and check-out function on the mVBR-EI app on their smart phones or tablets, thereby confirming that the home visit occurred and recording the time that was spent providing therapy. Similarly, check-in and check-out occurred on the app when a child visited a center for therapy. Program engagement rates were therefore informed by the ability of both the service provider and the families to attend appointments.

Mean program engagement was calculated as a percentage during 5 different assessments intervals (T1 to T5), with each interval encompassing a 6-month time period. The exception was T1 which encompassed 4-month period starting April 2017. In addition, the mVBR-EI app's dashboard allowed for various reports to be generated including program engagement for individual children, CRWs, specialists, blocks, and EI centers. These measurements allowed for interventions at a micro (individual child or service provider) and meso (program) level to improve program engagement.

To identify engagement categories, the team reviewed the distribution of engagement to identify natural cut points while considering clinically meaningful exposure. Based on these considerations the following three program engagement groups were chosen:

- </= 60% = low engagement
- 61–80% = medium engagement
- >/= 81% = high engagement.

Of the 1,136 children that were eligible to participate in the study, a total of 1,050 children's (679 boys and 371 girls) families consented to participate and they provided 3,408 program engagement measurements over the course of the study. A total of 274 children withdrew from the study; 78 children were discharged due to significant improvement or being transferred to a school therapy program and 196 children withdrew for other reasons, with the most common reasons being families moved out of catchment areas (*N* = 113) and parental withdrawal (*N* = 40).

*School enrollment: definition and calculation.* In Tamil Nadu, children are able to enroll in school after their third birthday. Therefore, all children aged 3 years and above at the time of assessment, whose primary caregiver was available to be asked about school enrollment and who consented to participate in the study were included. Eligibility for school was determined at each assessment round.

School enrollment data was first collected in July 2017, and then every 6 months after for a total of five measurements (T1 to T5). School enrollment was determined by caregiver response to the question about whether or not their child was currently enrolled in school by a rehabilitation specialist. Type of school (if enrolled) and reasons for non-enrollment and school dropout were also recorded. To reduce bias, specialists were blinded to the participant's previous responses about school enrollment and were not the child's treating specialists.

#### Results: Focus Group 2 and Interviews

**Figure 4:**
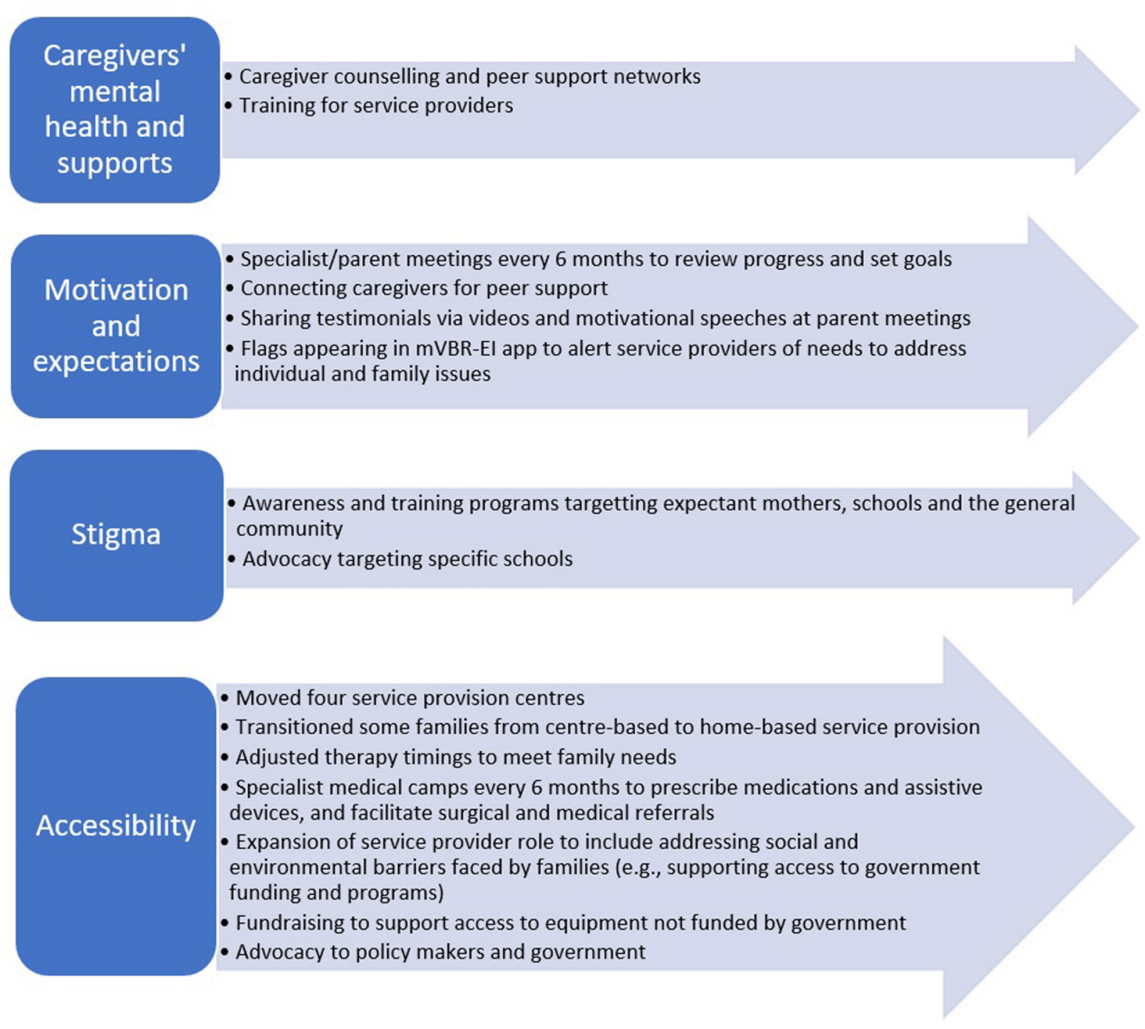
Summary of program changes implemented in Case 2 that addressed aspects impacting program engagement and school enrollment.

Figure 4 summarizes the findings of this case. While separate sets of questions were asked of caregivers and service providers about their thoughts on program engagement and school enrollment, we found the following themes impacted both aspects: (1) Caregivers' Mental Health and Supports, (2) Motivation and Expectations, (3) Stigma, and (4) Accessibility. As such, qualitative results for these two aspects are presented together.

##### Caregivers' Mental Health and Supports

Caregivers and service providers shared experiences where caregivers' mental health and access to supports influenced engagement in therapy and school enrollment. Caregivers with increased strain, stress, depression and poor mental health had lower levels of therapy engagement and school enrollment according to service providers and caregivers themselves. A need for greater psychological support for these caregivers was highlighted through this study.

Access to supports also influenced appointment and school attendance. Some families were encouraged to attend school and EI therapy regularly by their medical doctor, ASSA service providers and/or supportive family and community members. When some mothers, who are the primary caregiver, are unable to attend the child's appointment or take them to school, they have support from other family members or friends who bring the child to their appointment and school or are available at home to greet the service providers. In other cases, family and community members provide financial or caregiving support to caregivers.

In contrast to this, some caregivers stated that lack of support for them within the family, and lack of community networks led them to be the sole support for their child with a disability. This led to poor program engagement and lower school enrollment as the child was completely dependent on one or two caregivers, and if they were not available due to sickness, other child care needs or work commitments, their child missed out on therapy appointments or going to school. Caregivers and service providers highlighted a need for greater peer support networks in the community and among family members of children with disabilities in order to increase both practical and psychological support for them.

The level of support from staff at local schools played a critical role in families' abilities and willingness to enroll their children with disabilities. Some caregivers shared that teachers or headmasters encouraged them to enroll their child, through a graduated integration (e.g., starting with once a week and then increasing) and by providing supports such as peer helpers. There are also many supports available to children attending schools provided by the government that include financial support for transportation, personal support workers in school, therapy in school, assistive devices, and home tutoring support. However, knowledge and access to such supports varied considerably amongst caregivers. To receive this support, it often required the caregiver self-identifying a need and advocating on their child's behalf which was often a challenge for many. If these supports are not offered by the school or accessed by caregivers, caregiver soften did not send their child to school because they felt they can provide a better quality of life for their child at home.

##### Motivation and Expectations

The primary motivator for high program engagement was seeing improvement in the development of their child. Caregivers stated they were more likely to attend therapy regularly if they perceived improvements in their child and felt that therapy was leading to that improvement and/or that therapy was meeting their goals and expectations. This led to feeling hopeful about their child's future. One mother shared:

“*When we started at the center, I saw improvements, which motivated us to continue coming regularly because it reduced my burden and stress”* [interpreter translation from Tamil].

Some caregivers shared that when they did not see improvements, attending therapy sessions was less of a priority for them. Service providers stated that caregivers with realistic expectations around their child's prognosis had higher program engagement rates compared to those expecting changes that were unrealistic or who had unrealistic timelines. They also stated that caregivers with greater understanding and knowledge surrounding the nature of their child's disabilities and abilities and the purpose of early intervention therapy had higher program engagement rates. Service providers felt that educating caregivers on their child's disabilities, abilities, prognosis and purpose of early intervention therapy was a very important factor in improving their level of program engagement.

Most caregivers were highly motivated to have their child attend school and considered school as an opportunity for their child to learn skills and gain a livelihood later on because their caregivers would not be able to support them forever. School was seen as one way that children could become independent by gaining academic and social skills. One caregiver shared:

“*If I keep my child out of school, he will not be aware of the outside world*.

*It is important that he gains knowledge, even just basic knowledge, in order to be able to navigate in the community, like take public transportation”* [interpreter translation from Tamil].

Specialists added to this theme, highlighting that some children learn academic skills while others may not necessarily learn to read or write, but they learn how to sit for 2 hours which is seen as a big accomplishment by caregivers. Specialists also added that enrolling a child in school could provide some respite for caregivers. Caregivers' motivation for enrolling their children in school also revolved around their desire for achieving “normality”. Some caregivers felt that it was best to enroll their child in a mainstream integrated school so their child could learn how to do things like typically developing children.

Other caregivers felt that early intervention therapy was the main factor that allowed for child's development to improve enough to have them enroll and integrate into school. For example, one child had tried attending school but had difficulty with feeding and writing. He took a break from school to receive therapy for a year, focused on acquiring both those functional tasks, and once achieved, was able to be integrated into school. Caregivers expressed their desire for EI therapy and school to be more complementary in order to achieve academic, social, and other developmental goals. Service providers highlighted that caregivers often need to be encouraged and motivated to enroll their children in school, as fear and stigma were often barriers.

##### Stigma

Family, neighborhood, school and community stigma were stated to influence program engagement and school enrollment. Some caregivers and service providers shared that stigma often involved blaming mothers for the child's disabilities, gossip about the family or being shunned by neighbors. This led some caregivers to want to hide their child's disability and not be seen going to therapy centers, schools or have service providers entering their homes. A service provider shared the following story:

“*When the specialist arrived for the initial visit with the family, the family was asked by neighbors why specialists were visiting their home. When the caregivers explained why, the neighbor stated that the child would be delayed for marriage because of therapy. The neighbor said this loudly in the middle of the street. The caregivers stopped treatment”* [interpreter translation from Tamil].

According to both caregivers and service providers, stigma played a major role in a child's ability to enroll and integrate into school and was found to occur at the level of peers, teachers and school administrators. Peers often excluded children with disabilities from play and activities. Children were sometimes teased and called names by their peers because of their disability. Children were interested and motivated to attend school, but when they were teased, they no longer wanted to go to school. School peers often lacked understanding of the child's ability and how to modify play and activities to make it safe and enjoyable for children with disabilities.

School attitude and stigma about disability and integration seemed to greatly influence the capacity for a child with special needs to be integrated in school. Caregivers stated that teachers and school administration often encouraged them not to admit their child to that school because they did not feel that they were capable and would instead recommend they attend a special school or receive home schooling. Service providers shared that school administrators felt that children with special needs may bring down test scores or have higher dropout rates at the school and this would reflect poorly on the school. One caregiver expressed frustration:

“*If my child was sent to a special school, she would not be integrated in the normal society. But, when she is going to regular school for integration, my child is excluded from the group because of her disability”* [interpreter translation from Tamil].

Community stigma played a major role. Interviews revealed that caregivers of other children complained that their children would adopt the behaviors of children with disabilities and encouraged headmasters and teachers to separate or exclude children with disabilities from school activities or school enrollment in general. Though all children have a legal mandate under India's Education for All Act, perceived and actual stigma led to challenges in school integration and discouraged caregivers from enrolling their children into school.

##### Accessibility

Program engagement and school enrollment were affected by a family's ability to access early intervention or school services which included transportation, family income, child factors, physical environment and access to assistive devices, and accommodations in school. The lack of accessible public transportation and cost of private transportation (e.g., personal vehicle, taxi) was a major barrier to bringing children to center-based therapy appointments and school. In the study area, most children walk to school or are taken on motorcycles. As such, child mobility issues and physical disabilities were major barriers to using these modes of transportation. Some schools do provide school bus services, but they were usually inaccessible as the child needed to be brought to a bus stop and buses were not modified to accommodate children with physical disabilities.

Family income level was also noted as a barrier, even if therapy services are provided at home. Since families need to be present for both home-based and center-based therapy, work opportunities were noted by some to be prioritized over child therapy visits, impacting their ability to engage in the therapy program. However, this was less of an impact for home-based therapy as visits were once a week compared to three times per week for center-based therapy. Finally, home services were seen as beneficial because service providers use equipment already at home and therapy was provided in the setting children spend most of their time in. Therefore, caregivers learned to integrate therapy into the child's daily life in a more “real” environment, outside of their weekly session with service providers.

Child factors, including type and severity of their child's disability could be a barrier for caregivers to take them to EI centers and schools. One example was a caregiver stating she could not take her son to school because he was not an independent walker and was too heavy to be carried. Many caregivers shared bluntly that if their child cannot walk, their child cannot attend school. Distance from their homes to the EI centers and schools were also noted as related barriers. Home-based therapy was seen as more accessible by caregivers as it overcame some of these barriers. Aside from walking, independence in activities of daily living, especially toileting, was also listed as an important facilitator to attending school. If the child was unable to effectively communicate their need for toileting or ambulate independently to the toilet, caregivers did not feel comfortable sending their child to school or the school discouraged their enrollment.

A child's severity of disability and medical co-morbidities played a large role in both program engagement and school enrollment. A child who had little to no functional impairments could integrate into school with ease and little adaptation was required from the school. However, children with greater severity of functional impairments had lower school enrollment. Child illness was noted as a factor for missed therapy appointments. The physical environment at school was also listed as an important factor. Ramps, rails and accessible toilets for children with mobility challenges were not available at most schools. Children who had access to schools with a barrier-free environment were more likely to be enrolled. Caregivers highlighted that the lack of accessibility made them hesitant about the ability of the school to care for their child effectively.

Many caregivers noted that inability to access assistive devices such as wheelchairs, walkers, hearing aids, braille textbooks, etc. were barriers to school enrollment and integration. A lack of accommodation in curriculum delivery to children with disabilities and varying learning needs was highlighted as a major barrier to school enrollment. In the study region, resources in government schools consists of one special educator covering 15 schools and providing consultation and advice to teachers on adapting the curriculum to children with special needs in their class. Many service providers and caregivers interviewed felt that these resources were not sufficient to meet the needs of children with disabilities. Another barrier identified by caregivers was a lack of access to medical specialists who could prescribe medications to their children to control behavior, seizures or spasticity, all factors that could impact program engagement and school enrollment and integration.

Although inclusive education is government-mandated in India, service providers shared that little is done to make this possible. Teachers have classrooms of 40 or more children and do not have the knowledge and time to modify education to the child's needs. When children with a mild or moderate cognitive delay were enrolled in school, teachers often discouraged caregivers, stating that they cannot teach and take care of the child and the child will not be able to adapt to the school environment.

#### Rapid Cycle Evaluation Action Taken

The data collected through interviews and focus groups was complemented by qualitative and quantitative data collected with individual children and families. As described in section Program Design, development scores, family outcomes, and caregiver feedback were collected every 6 months for all children and families in the program. Based on all these data sources, a number of rapid cycle actions were taken throughout the course of the study and are listed under the theme categories described above.

##### Caregiver Mental Health and Support

To address the issues of caregiver mental health and support, and to improve program engagement and school enrollment rates, counseling, and peer support networks were introduced. A psychologist was hired and families who had low program engagement, high levels of strain and low family empowerment were flagged on the mVBR-EI app dashboard and were referred for counseling. Counseling occurred in both one-on-one sessions and in group sessions. These sessions focused on caregivers' mental health and increasing their supports. CRWs and rehabilitation specialists were also advised by the psychologist on how to better support caregivers' mental health during their regular therapy visits.

In addition, peer networks were created in the form of eight Early Intervention Parent Participation groups (one for each block). Parent leaders were elected and monthly group meeting at the center were held between caregivers and social media networking groups through WhatsApp were formed between caregivers. In addition, large group meetings for parent consultations and networking were organized every 6 months, where all caregivers and family members involved in the program were invited. These opportunities allowed caregivers to share, interact, and form social support networks. Some caregivers formed transportation-sharing networks to help get their children to school or EI center appointments. Some caregivers started joint small business enterprises to financially support each other. Other caregivers started having their own small group meetings and organized play dates with their children. These parent groups also acted as a collective force to advocate for school enrollment and accommodation of their children with disabilities.

##### Motivation and Expectations

To address the issue of motivation and expectations, a policy was created whereby each rehabilitation specialist reviewed the developmental scores of a child on the mVBR-EI app with their caregivers every 6 months. During these visits, prior to goal setting, specialists highlighted the progress of the child to date, which helped caregivers recognize subtle gains and motivate them toward greater engagement in therapy. In addition, the anticipated functional outcomes were highlighted to set realistic expectations. Caregivers and service providers were encouraged to set therapy goals and priorities that would help achieve school enrollment and integration.

If caregivers showed a lack of motivation or unrealistic expectations, they were connected to a family of a child with similar disability for peer support, role-modeling and motivation. Feedback and testimonial videos of other children and their families were developed and shared with caregivers. In addition, during parent meetings, motivational speeches were given by caregivers in the programs, village leaders and youth with disabilities to motivate caregivers toward greater EI therapy program engagement and to enroll their children into school.

##### Stigma

To address stigma, awareness and training programs were instituted for the community and delivered by CRWs and specialists working in the mVBR-EI program. Three categories of awareness programs were formed: Women, Schools and General Community. Women's Awareness programs were designed for expectant mothers and General Community Awareness programs were for anyone living in the community. The programs sought to increase awareness about early childhood development, childhood disabilities, early intervention therapy, the potential of children with disabilities and how to better integrate children with disabilities in the community and support families. These programs were done with the involvement and support of local community leaders with the goal of reducing stigma and allowing the community to understand the aim of our early intervention program. A total of 20,417 participants attended these programs between April 2017 and August 2020.

The school awareness programs were conducted at primary and secondary schools and focused on raising awareness about childhood disabilities, the rights and laws related to school enrollment for all children and how to accommodate and integrate children with disabilities into the classroom and school setting. These programs were attended by a total of 13,338 students, teachers and school administrators over the 28-month study period. Pre-school teacher training programs conducted by ASSA's service providers focused on training for identification of children with delayed development. These training programs were strengthened to include how to integrate, accommodate and teach children with delayed development in pre-schools. In total, 2,033 pre-school teachers were given this enhanced training. In addition to these awareness and training programs, advocacy at individual schools occurred. If a child was denied school enrollment or proper accommodations were not given, advocacy by that child's CRW and rehabilitation specialists and EI parent groups occurred to facilitate enrollment and accommodation.

##### Accessibility

**Figure 5:**
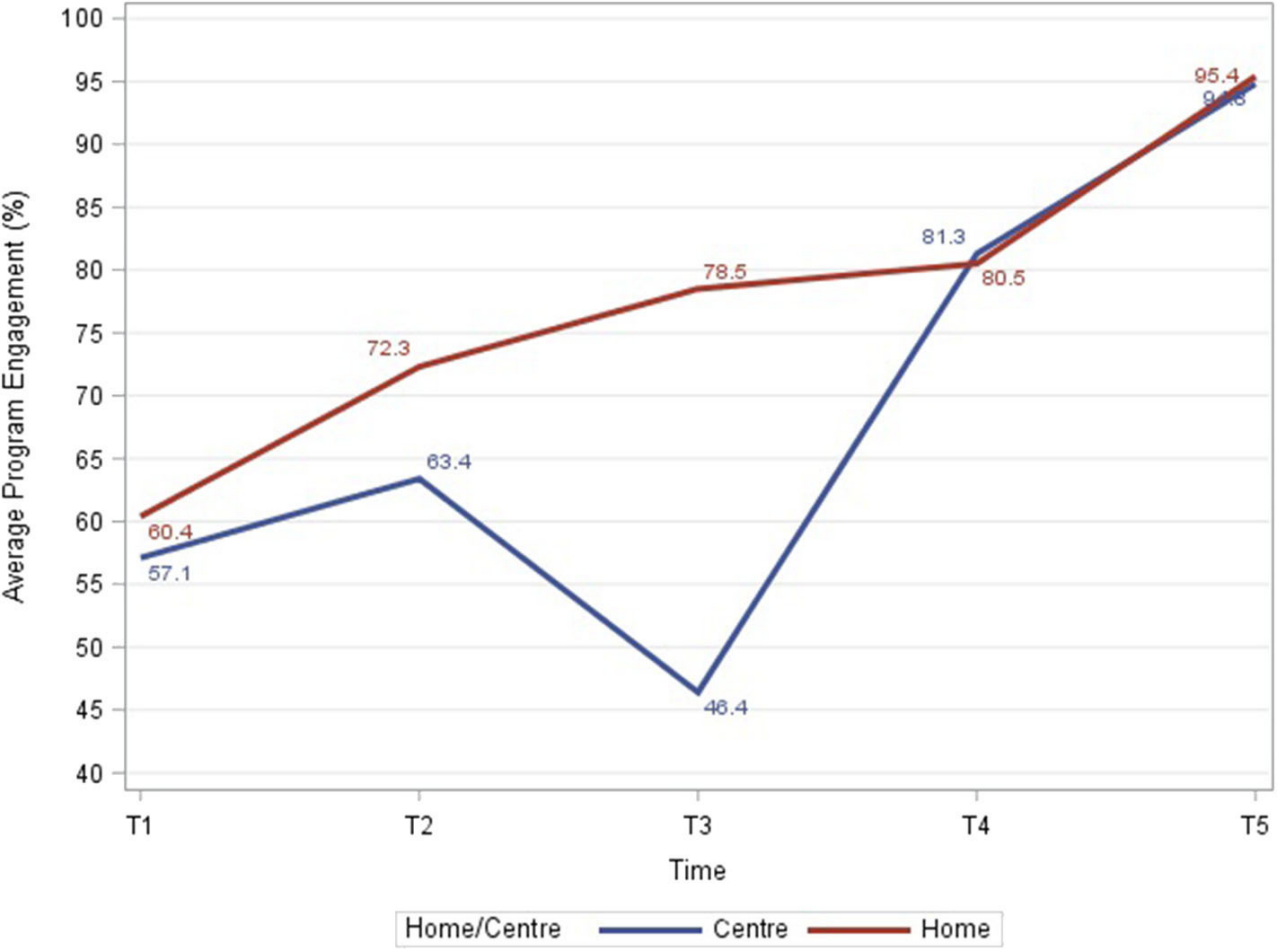
Average program engagement rates for center-based and home-based programs during each time interval.

At T3, there was a drop in program engagement rates for center-based children from 63 to 46% (see Figure 5). The mVBR-EI dashboard was examined and it was revealed that the drop-off was largely driven by four out of the eight EI centers. The management team consulted service providers and families working and attending these centers. They revealed that the main issue was that the public transit bus route had been changed and the location of the centers were no longer close to bus routes. This caused many families to miss appointments or stop coming altogether. Solutions were sought to this issue, including ride sharing and van service. However, they were not feasible and these four centers were re-located. In addition, 22 families with low program engagement accepted the opportunity to transition to the home-based program. Management redeployed staffing of service providers to meet this adjustment.

To help families work around competing priorities, including work and other childcare needs, schedules were established in consultation with individual families to adjust therapy timings to their needs. In addition, to prevent families from having competing priorities of attending therapy vs. school, therapy sessions were scheduled in school during school hours. This had the added positive effect of connecting our service providers with teachers. This helped build positive relationships and provided an opportunity to educate teachers on how to better accommodate, integrate and provide therapeutic activities for children with disabilities in their classrooms.

To meet the need for increased access to assistive devices that was identified in the stakeholder consultation, a survey of existing community programs and resources was undertaken. It was recognized that some equipment is funded through government programs, but often families were not able to access medical professionals to obtain prescriptions for the required corrective equipment. In addition, it was recognized that some of the needed equipment, including pediatric wheelchairs, were not funded. To address these issues, ASSA set up medical camps. Medical camps were organized every 6 months where medical and surgical specialists were brought to one convenient location and children in the mVBR-EI program were referred to them by CRWs and rehab specialists in our program. The medical specialists included orthopedic surgeons, pediatricians, urologists, neurologists, neurosurgeons, ophthalmologists, otolaryngologists, speech, and hearing specialists and dentists. These specialists prescribed needed medications, adaptive equipment and facilitated further surgical and medical referrals to government and private hospitals. CRWs also helped families' access available government funding for equipment.

In addition, funds were raised from donors to set up an equipment provision fund in which service providers could apply on behalf of families for funding for adaptive equipment not covered by government programs. To further improve school enrollment, service providers also started to help families access other government funding available, including transportation support, school personal support worker services, and home tutoring support. Thus, there was an expansion of the role of service providers to include addressing social and environmental barriers faced by families.

#### Indications of Effectiveness

Through the longitudinal cohort study, it was possible to monitor levels of program engagement and school enrollment. The trends over the duration of this study were largely positive, with increased program engagement and school enrollment over time. These changes could be a result of the rapid cycle actions evaluation taken. However, given the study design, the data below are not intended to reflect the causal impact of the changes implemented. Rather, the longitudinal data has been used to monitor changes and serve as a feedback loop of data for the changes implemented.

##### Program Engagement

At completion of the first interval, T1 (0–4 months), the overall program engagement rate was 59.9% and this gradually increased over the next four time periods to 95.3% by T5 (22–28 months). Center-based program engagement rates started at 57% and dropped to 46% at T3 (11–16 months) and then increased to 95% by T5 (22–28 months) while home-based program engagement rates steadily increased with time (Figure 5).

**Figure 6:**
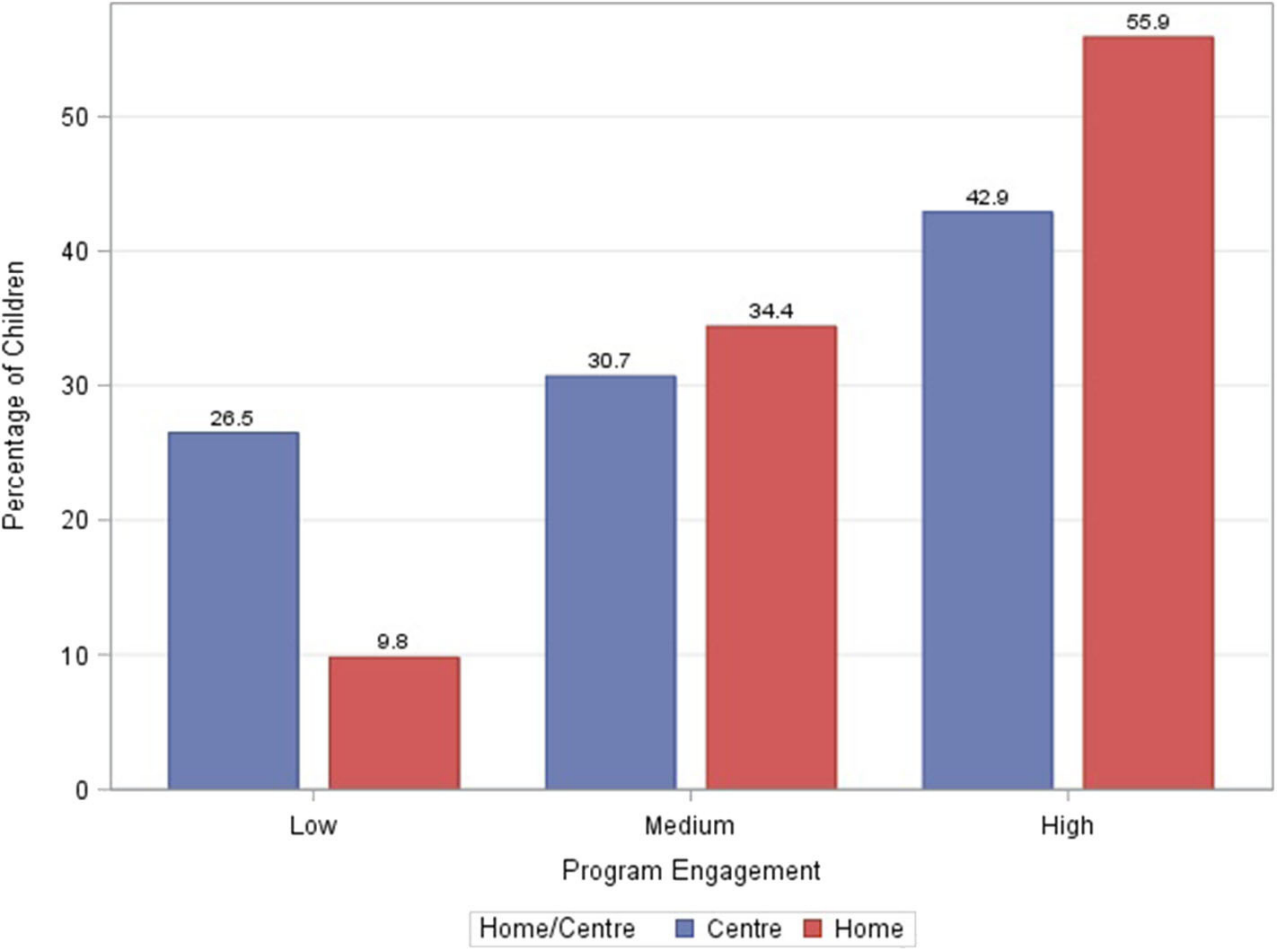
Cumulative program engagement over the duration of the program segregated by low (</= 60%), medium (61–80%), and high (>/= 81%) rates of engagement for center-based and home-based programs.

**Figure 7:**
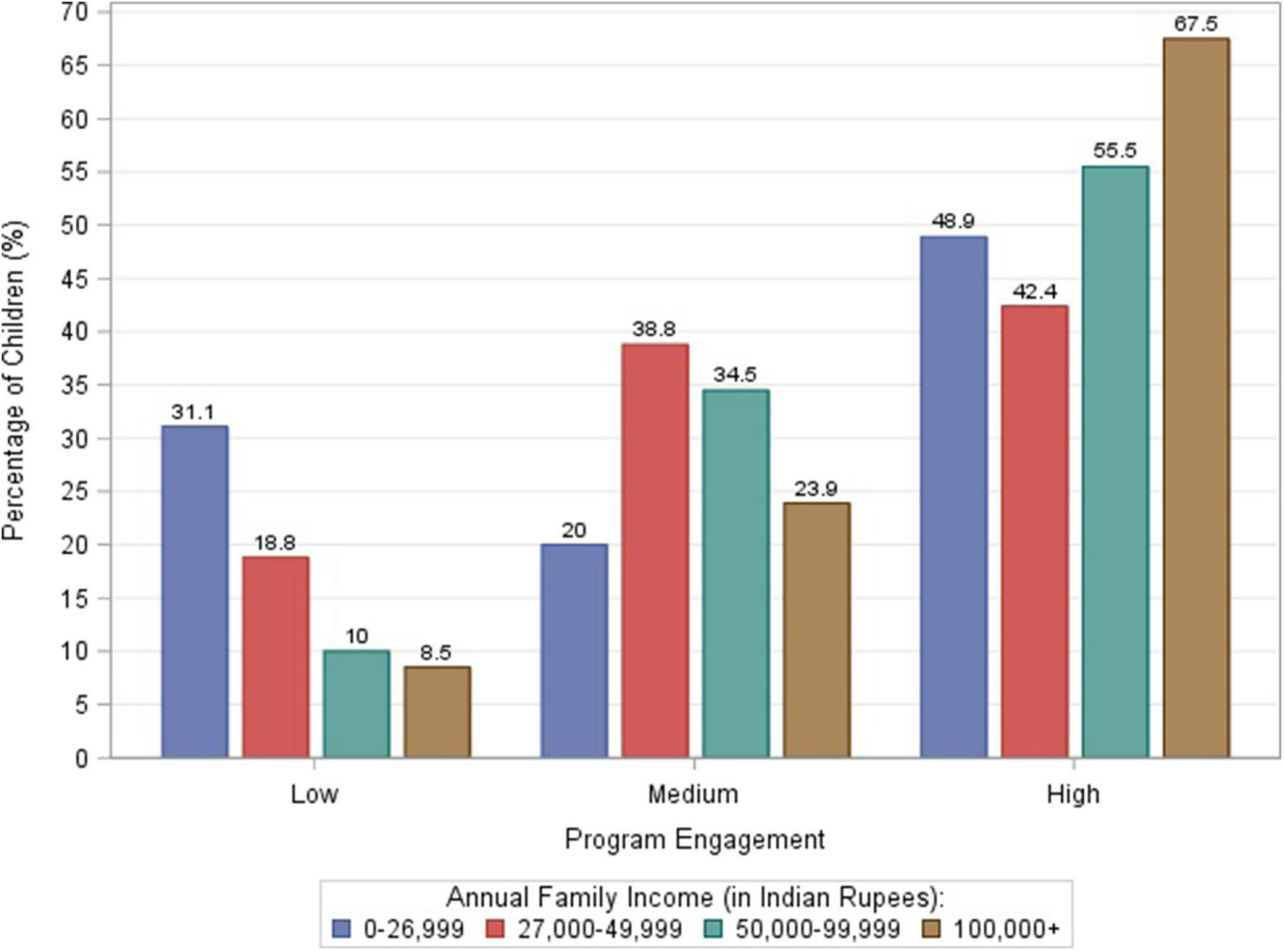
Cumulative program engagement over the duration of the program segregated by low (</= 60%), medium (61–80%), and high (>/= 81%) rates of engagement for various family income levels.

Overall, 53.5% of children had high engagement (81–100%), 33.7% had medium engagement (61–80%), and 12.8% had low engagement (0–60%). The distribution of center-based vs. home-based by program engagement group was significantly different with higher program engagement among children receiving home-based therapy, cumulatively from T1 to T5 (*p* < 0.0001; Figure 6). There were no statistically significant differences in program engagement by gender. The distribution of family income by program engagement was significantly different with higher engagement among families with higher income (*p* < 0.0001; Figure 7). The distribution of program engagement groups by type of disabilities (*p* = 0.08) and severity (GMFCS levels) for children with cerebral palsy did not show any statistically significant differences (*p* = 0.686).

##### School Enrollment

**Figure 8:**
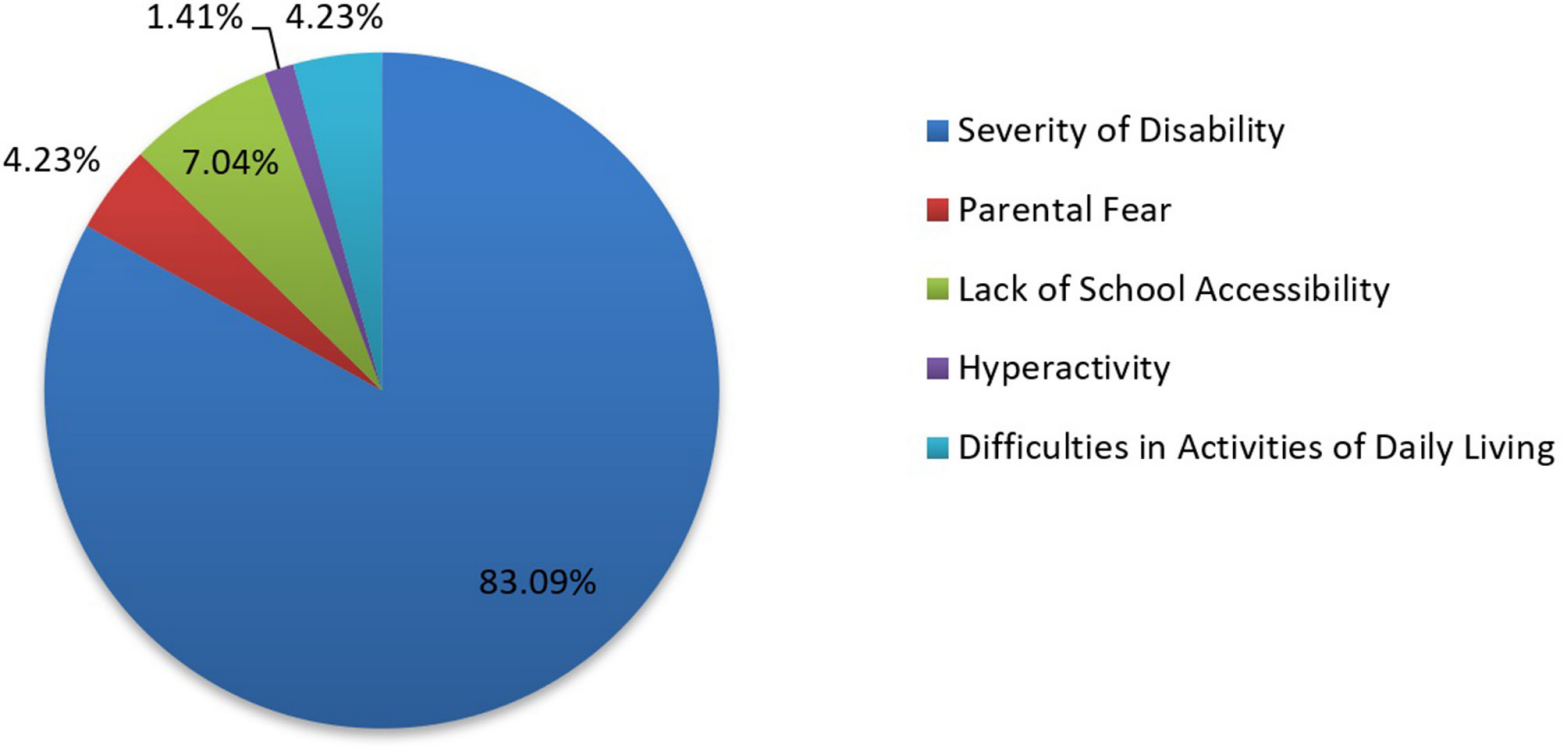
Distribution of children not enrolled in school by reason for non-enrollment at T5.

The proportion of children enrolled in school at each of the assessment points increased over time from 69.8% at T1 to 84.7% at T5. There were no statistically significant gender or age differences in school enrollment. There was a statistically significant difference for school enrollment by location of therapy (*p* < 0.0001) with higher enrollment among children receiving home-based therapy. At T5, the primary reason for children not attending school is shown in Figure 8. At T5, among children enrolled in school: 68% were in primary school, 27% were in pre-school, and 6% were in a special school.

**Figure 9:**
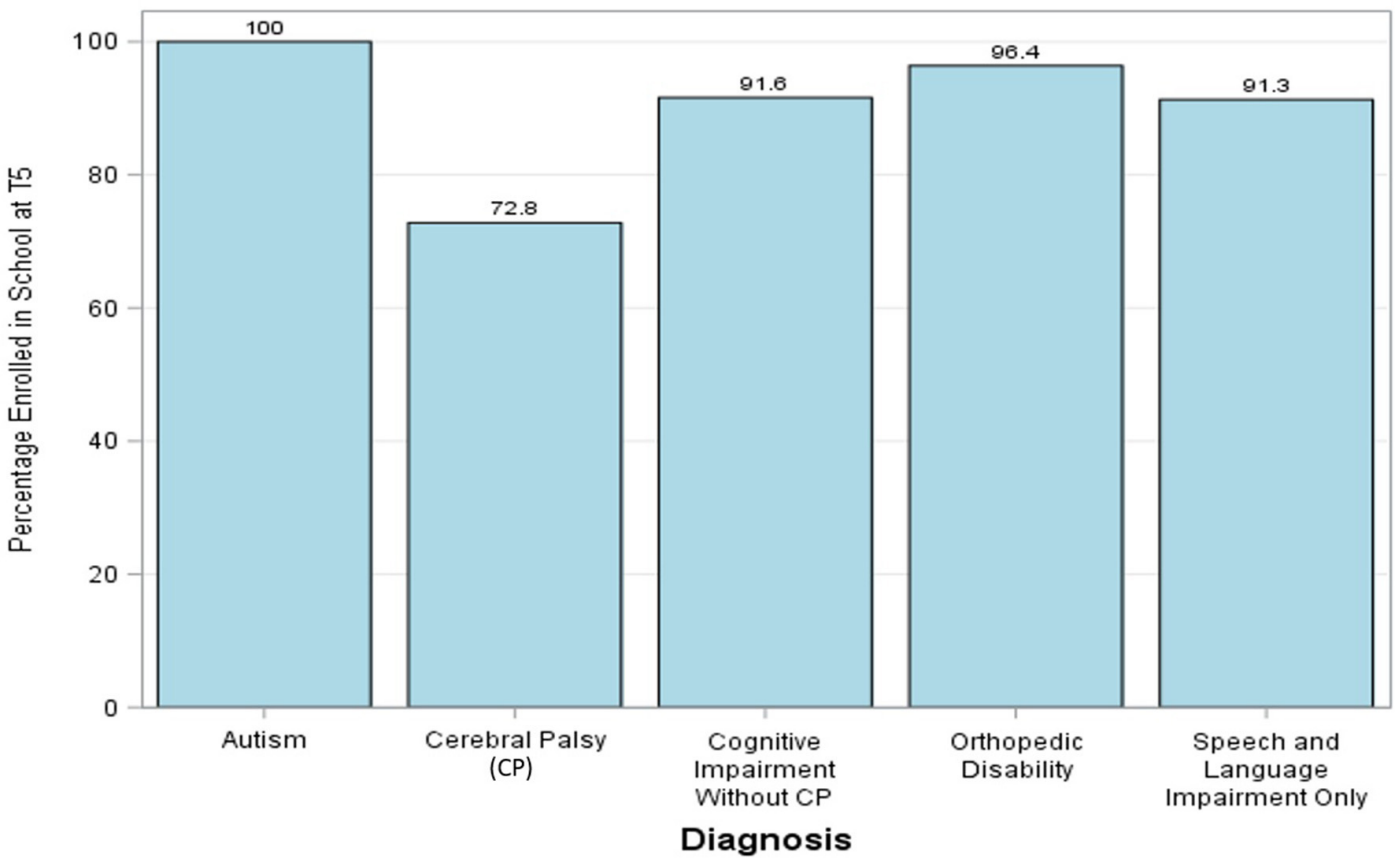
Percentage of children with various diagnoses enrolled in school at T5.

**Figure 10:**
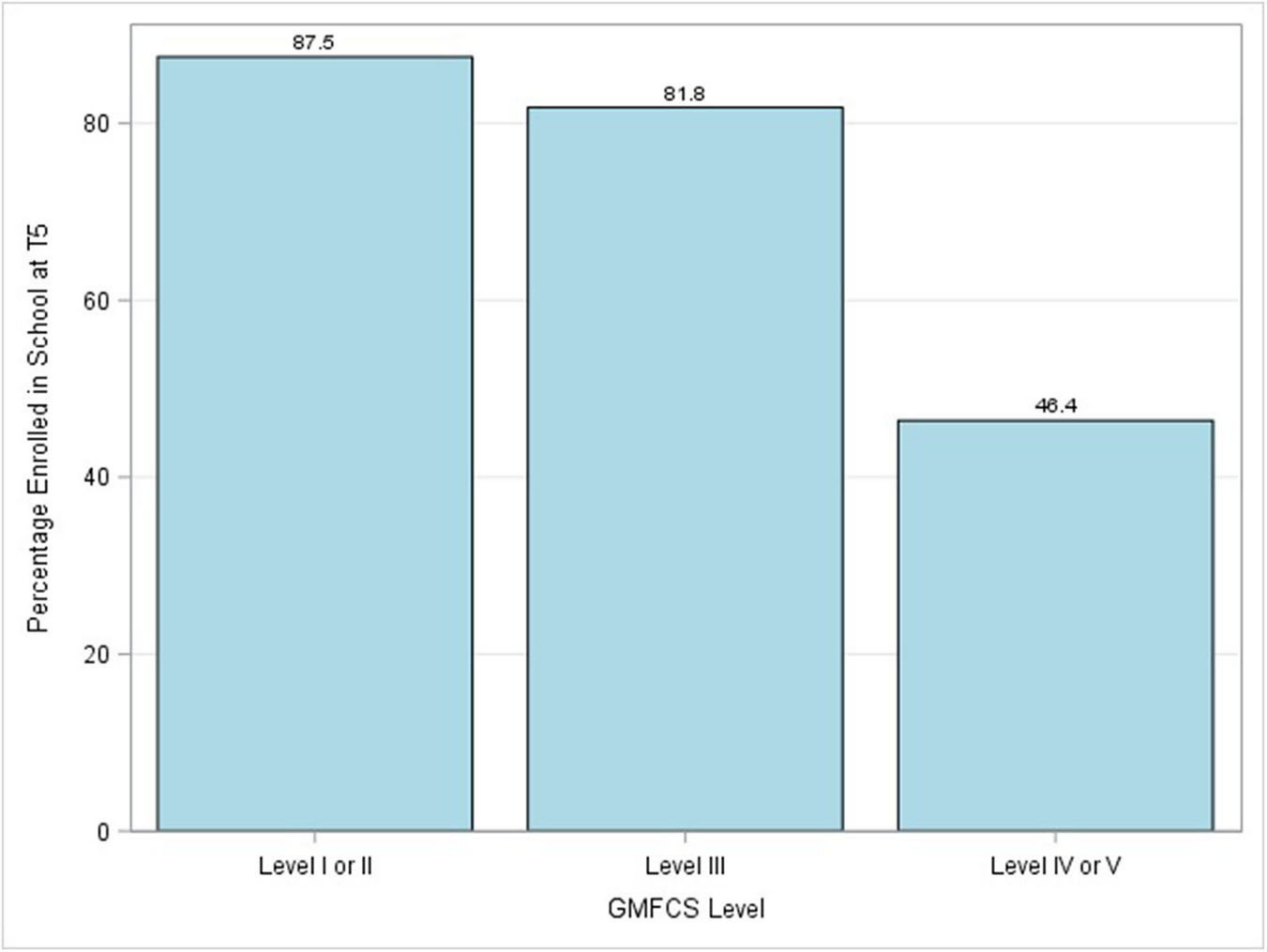
Percentage of children with cerebral palsy with various levels of severity as defined by GMFCS levels enrolled in school at T5.

At baseline, 132 children (41 girls and 91 boys) were not enrolled in school and were still in the study at T5. Of these children, 63 (47%) achieved school enrollment at T5. At baseline, 335 children were enrolled in school and 97.9% were still enrolled in school at T5. Enrollment by type of disability at T5 varied from 72.8% for children for cerebral palsy to 100% for children diagnosed with autism (Figure 9). Children with cerebral palsy with greater severity (i.e., GMFCS Level IV or V) were less likely to be enrolled in school (Figure 10).

## Discussion

### Program Outcomes

Our unique theoretical perspective combined the biopsychosocial framework of ICF along with a CBR strategy and resulted in successful rapid-cycle evaluation and program improvements.

The ICF integrates relevant information on the different components for individual, environmental and personal factors to guide interventions and inform program planning, monitoring and evaluation (11). This holistic picture proved to be one of the major benefits of using the ICF to guide our CBR program.

Measurements, involving stakeholder consultation, led to action that had an effect on improving working conditions for service providers. This resulted in improved service provider well-being during work, including improved health, access to and use of bathrooms and sense of safety. However, societal barriers including menstrual stigma and lack of bathrooms facilities remained challenges to service providers. These require broader interventions including policy-maker advocacy to provide bathroom facilities in homes and community spaces in rural areas as well as improved societal awareness to address and reduce menstrual stigma. ASSA recognizes that there may be limits to our ability to fully impact societal barriers, which are deeply ingrained, but remain committed to modeling, promoting and advocating for inclusive policies.

Program engagement and school enrollment both increased significantly over time. It is possible that the increase in school enrollment was because the cohort of children aged 3 years and above got older over time. However, there was no statistically significant difference in school enrollment based on age at T5, so this is likely not a major factor for the increase in school enrollment seen.

Addressing service providers' quality of work-life and challenges in reaching children's homes through education of health and resource mapping likely had a major impact in improving program engagement (i.e., visit attendance) over time. Improving caregiver mental health through psychological counseling, peer support networks and training service providers to motivate and set realistic expectations with families likely had an impact on improving program engagement and school enrollment as well. Having identified family, school and community stigma as a major issue through consultation and conducting awareness program targeted at reducing stigma likely also contributed to positive changes. This coupled with training programs for pre-school teachers and advocacy at schools likely had an effect on improving school enrollment and program engagement, as we observed a shift in the community's attitude toward children with disabilities and their potential.

A family-centered approach was supported by considering the contextual factors related to child, family, services, and culture (33). Stakeholder consultation revealed caregivers faced a lack of access to medical consultations, adaptive equipment, and medications. By responding with a mechanism to provide increased access to adaptive equipment such as wheelchairs, walkers, orthotics, hearing aids and eye glasses and medications for medical comorbidities such as seizures, spasticity and hyperactivity, it likely had a major effect on improving child well-being and function that facilitated improved program engagement and school enrollment. Similarly, access to government grants and supports including transportation assistance and building family networks for ride sharing greatly assisted families in taking their children to school and therapy.

The actions described were taken at a meso level (program), but the monitoring mechanisms allowed for measurement at a micro level (each child, family, or service provider). Families with high strain or low empowerment, children not meeting expected development milestones or children not enrolled in school, all created flags on the mVBR-EI dashboard. These flags were then reviewed by those families' service providers, field team leaders and management and targeted action was taken. This rapid cycle action included providing psychological counseling to caregivers, providing peer mentorship, adjusting individual therapy goals and plans, referral to medical camps, provision of adaptive equipment and advocating at individual schools for enrollment, accommodation, and integration.

The qualitative measurements complemented the quantitative measurements in our rapid cycle actions. These data revealed that children with a primary motor disability, such as cerebral palsy were less likely to be enrolled in school compared to children with other disabilities, including speech and language disabilities. In addition, those with greater severity (higher GMFCS levels) were less likely to be enrolled in school than those with more minor motor impairments (lower GMFCS levels). This was coupled with the results of the focus groups and interviews that revealed a lack of physical accommodations in school (i.e., ramps, rails, accessible toilets) and that caregivers were unlikely to enroll their child in school if they could not walk. This led us to focus on advocacy for greater physical accommodations at individual schools. In addition, we recognize that advocacy with policy makers to ensure accommodations set out in the Education for All Act are universally fulfilled are also needed. A barrier identified for program engagement was the service access inequity along income lines. Changes in this domain would require government policies and public support for initiatives such as improved minimum wages, and employee benefits such as paid time for caregiver responsibilities. Raising these inequities is part of public education that supports broader social movements toward such changes.

### Study Limitations

A limitation of this study is that rapid cycle actions were bundled, multi-pronged interventions and therefore, we are unable to determine the effect-size of any one intervention. For example, did school enrollment increase more because of advocacy at individual level or because of school awareness programs? Did program engagement improve more because of parent group formation or because of addressing service provider work challenges? Such questions cannot be answered with the data collected in this study. Another limitation of this study for the quantitative data analysis is a lack of a control group, blinding and randomization.

Another limitation is that only 14 families of 1,050 families in the study participated in focus group 2 that explored barriers and facilitators to program engagement and school enrollment. Therefore, it is possible that other perspectives were not captured. However, purposeful sampling of families aimed to represent a broad cross-section of participants, including low, medium, and high program engagement and school enrollment and non-enrollment were chosen and by the end of data collection, similar themes were recurring in interviews. In addition, ongoing administrative review of feedback from families throughout the study using the Tirunelveli Early Intervention Caregiver Assessment Tool confirmed and supported the themes presented.

### Experience With Rapid Cycle Evaluation

#### Cost and Opportunity

The study incurred a range of direct costs to set up, such as hiring research consultants, interviewers, and translators. In addition, as the study worked with a range of stakeholders within the program, there was the indirect cost of not providing service to children and caregivers while service providers were involved in focus groups and interviews. As the interviewers were intentionally selected to be external to the program, for reasons of objectivity and validity in data collection, it also took more time for study participants to build rapport with interviewers than if they had been drawn from program staff.

However, the data collection and analysis approach also generated a range of opportunities. First, through the Rapid Cycle Evaluation, there was a real feedback loop between data being collected and modifications being made to the implementation of the program. This relatively quick and visible link between data collection and implementation meant that the hesitation that was felt by service providers as well as caregivers to take part in data collection was reduced. This was evidenced by an increase in the number of service providers wanting to take part in interviews and focus groups over the duration of the study. Service providers and caregivers also voiced that they felt empowered and had a strong voice in the co-creation of the program, as their feedback led to visible programmatic additions and changes.

Second, having formalized data collection and feedback through a study setting, the data and insights generated carried more weight and credibility. The data from the study, rigorously collected and analyzed, enabled a well-informed conversation between ASSA management and Tamil Nadu state government given the credibility of the data. This in turn, proved extremely valuable in conversations with the state government regarding the scale-up of the program.

Third, the study approach provided a way to operationalize the inclusion of key stakeholders into the program. Many programs and interventions seek to organize the meaningful participation of stakeholders such as caregivers; but generally, this is not easy. Through the interviews and focus groups, it was possible to give a voice to both program staff as well as caregivers. As the Rapid Cycle Evaluation allowed for relatively quick implementation of some of the results, these data collection methods served as a concrete way to include stakeholders into the design and implementation of the program. Further, the qualitative data helped to explain results from the quantitative data. Fourth and most importantly, this study approach allowed for the flexibility of adapting without the confines of a more rigid study structure. This approach strengthened the delivery of the program which may have led to more positive outcomes for children and families.

#### Scale-Up

The monitoring and evaluation mechanism and ability for rapid cycle change put in place for this program has allowed the program to scale-up. The government of Tamil Nadu, in collaboration with Grand Challenges Canada, has agreed to fund an expansion of the program from the existing 8 blocks to 31 blocks. The Government of Tamil Nadu is also exploring the scale-up of this program methodology using the mVBR-EI app and monitoring system to all semi-urban and rural areas of Tamil Nadu with the goal of reaching 67,000 children with disabilities. Advocacy for this scale-up was driven by the high quality and fidelity data outputs and outcomes generated by ASSA. This, coupled with the flexibility and ability to institute rapid cycle action to improve program outcomes at both micro (with each child and family) and meso (program) levels including improved program engagement and school enrollment over time, provided policy makers in Tamil Nadu with the data needed to make an evidence-informed decision to scale-up the mVBR-EI program.

In conclusion, the process of using evidence-informed and stakeholder-driven adaptations in the early intervention program has led to greater program engagement, improved school enrollment, improved service provider quality of work life, and has successfully positioned the intervention for scale-up, providing lessons that may be beneficial in other contexts.

## Data Availability Statement

The raw data supporting the conclusions of this article will be made available by the authors, without undue reservation.

## Ethics Statement

The studies involving human participants were reviewed and approved the Health Sciences Research Ethics Board, University of Toronto, Toronto, Canada (ref #34653) and the Research and Development Committee at Kalasalingam Academy of Research and Education, Madurai, India. In addition, the study was registered with an international clinical trial registry, ClinicalTrials.gov (NCT03202966). Written informed consent to participate in the studies was provided by the participant or the participants' legal guardian/next of kin.

## Author Contributions

DK, SRS, SSM, ABh, RP, and ZC contributed to the conception and design of all components of the study. ABe, CA, MF, and LP contributed to conception, design, and data analysis of Case 1: Focus Group 1A. ABe, CA, SS, and AJ contributed to the conception, design, and data analysis of Case 1: Focus Group 1B and service provider questionnaire. ZC and JM contributed to the conception, design, and data analysis of Case 2: Focus Group 2 and Caregiver Interviews. BP and SM organized the database and facilitated the rapid cycle evaluation actions at the program level. ZC, LH, MB, JM, and JK conducted a literature review. DK wrote the first draft of the manuscript. ZC, JM, ABe, CA, MF, LP, SS, AJ, LH, MB, and JK wrote sections of the manuscript. All authors contributed to manuscript revision, read, and approved the submitted version.

## Conflict of Interest

The authors declare that the research was conducted in the absence of any commercial or financial relationships that could be construed as a potential conflict of interest.

## Supplementary Material

The Supplementary Material for this article, which includes interview guides and questionnaires, can be found online at: https://www.frontiersin.org/articles/10.3389/fpubh.2020.567907/full#supplementary-material

## Abbreviations

ASSA: Amar Seva Sangam
Com-DEALL: Communication Developmental Eclectic Approach to Language Learning Development Checklist
COPM: Canadian Occupational Performance Measure
CRW: Community Rehabilitation Worker
ECD: Early Childhood Development
EI: Early Intervention
FACP: Functional Assessment Checklist for Programming
FES: Family Empowerment Scale
GMFM: Gross Motor Function Measure
GMFCS: Gross Motor Function Classification System
MCSI: Modified Caregiver Strain Index
mVBR-EI: Mobile Village-Based Rehabilitation–Early Intervention
TDSC: Trivandrum Development Screening Chart
WHO: World Health Organization
WeeFIM: Functional Independence Measure for Children.
