# Supplementary material for: Rapid-Cycle Evaluation in an Early Intervention Program for Children With Developmental Disabilities in South India: Optimizing Service Providers' Quality of Work-Life, Family Program Engagement, and School Enrollment
Source: Front Public Health. 2020 Nov 30;8:567907. doi: 10.3389/fpubh.2020.567907 (PMC7734086; doi:10.3389/fpubh.2020.567907)
Supplement: Supplementary file 1 [file Data_Sheet_1.PDF]

Interview Guides for Focus Groups and Interviews and Questionnaire

Focus Group 1A - Service Providers Study on Gender, Work Quality of Life, Challenges and Opportunities

Guiding Questions for Focus Groups

Opening Questions:

- Can you tell us a little bit about your role as a Specialist/ CRW? How do you spend your work hours?
- Can you tell us a little bit about your life at home? Who are your family members (husband, children, parents, sibling)? What are your responsibilties?

General Experiences as Specialist/CRW:

- Share a positive experience that you have encountered while working as a CRW/Specialist.
  - How has being a woman played a part in this experience?
- Share a negative situation you have faced in your roles as a CRW/Specialist. How has being a woman played a role in this experience?
  Have you ever **felt** like giving up your job? Why? Why not?
- How do you think your experiences and your routine would be different if you were a male in this role?

Family/Community Support:

- Has your relationship with your family members (husband, mother-in-law, parents, siblings, etc.) changed as a result of your job?
  a. If so, how?
  b. What are they most pleased about?
  c. What are they most concerned about?
  d. if there are negative consequences, why do you still keep your job?
- What is your communities' (extended family / neighbours) perceptions of your role as a CRW?

Health:

- Does this job affect your mental and physical health? How?
- What is the main reason you take sick leave from work? How does this affect you?
- How does your menstrual cycle affect your work?
- How has pregnancy affected your role as a worker? Have you felt supported by the organization during this time?
- Does your job play a role in family planning? How? Do you participate in this decision?

Socioeconomic:

- What do you spend your earnings on? How does your house make decisions about finances (spending, saving)?
- Have there been any changes to your socioeconomic status as a result of your job?
- Have there been any changes in decision making about finances as an individual or family as a result of your job?

Organizational Procedures and Leadership:

- Have you noticed any differences in opportunities or treatment between men and women at Amar Seva Sangam?
  - If so what are they?
  - How do they affect your job as a CRW or Specialist?
- Are there opportunities for you to move up ranks in positions?
- If you are in a situation where you feel inequality, what resources have been provided to you from ASSA to help you?
  - How would you discuss your experiences with your supervisors?
  - What could be put in place to support you better?
  - How do you feel that EI project supports you as a female employee?

Safety

- How free and/or safe do you feel in moving around in the village and visiting people's home?
  - o How do you feel about moving around in the community as compared to before?
  - o How do you feel entering communities of a different culture or caste from you?
- What are the safety concerns related to your job? Related to being a female doing your job?
- If you feel unsafe during work, how do you respond?

Conclusion

- As a woman, is there anything you would want to change about your work, relationships, community?
- Is there anything else you would like to tell me about your job as a CRW / specialist and how it has impacted your family life, personal life and career goals?

Post-Intervention Questionnaire - Effectiveness of Strategies to address Gender-related Work Quality of Life and Work Challenges

**Job Title:**		**Gender:**	
**Work areas:**<br>**(blocks)**			
**Number of months worked at Amar Seva Sangam:**			

- Please rate your experience between **1 (no impact), 2 (minimal impact), 3 (moderate impact), 4 (strong impact)** and **5 (very strong impact)** for the statements below in areas of Health / Bathroom, Menstruation, and Safety.

**Health / Bathroom**	**1**	**2**	**3**	**4**	**5**
The health and hygiene training at ASSA has helped me improve my health during fieldwork.					
The health and hygiene manual has helped improve my health during fieldwork.					
The list of bathrooms in the manual has helped me access bathrooms during my workday in the field.					
The health and hygiene training at ASSA has made me feel less shy and more comfortable to ask families to use their bathroom.					
Any comments or suggestions on how to improve health or improved access to or attitudes around bathroom use during field-work?					

Menstruation	1	2	3	4	5
The health and hygiene training and manual made me feel it is okay to work or enter families' homes during my period.					
The health and hygiene training and manual has made me more mentally comfortable attending work when I am on my period.					
The health and hygiene training and manual has helped prepare me to attend work during my period in practical ways (e.g., find bathrooms, change, relieve pain)					
The health and hygiene training and manual helped me understand that there are many myths about working during menstruation.					
The health and hygiene training and manual made me realize that menstruation is normal and healthy part of female life.					
Any comments or suggestions on how to reduce menstrual stigma during field-work and in the community in general?					
**Safety**	**1**	**2**	**3**	**4**	**5**
The safety training and manual made me feel more safe going to villages and homes for my work.					
The safety training and manual made me more aware of "risk" situations related to my work.					
The safety training and manual made me know what steps I need to take when I do feel unsafe.					

The safety manual provided me with resources that made me feel more safe while working.					
Any comments or suggestions to improve your safety and sense of safety during field-work?					

Focus Group 1B - Service Providers Study Effectiveness of Strategies to address Gender-related Work Quality of Life and Work Challenges

Guiding Questions for Focus Groups

<u>Opening Questions:</u>

- Introductions: Name, Role, and how long have you been with Amar Seva Sangam?
- If you are new, what have your experiences been as a fieldworker?
- If you have been with Amar Seva Sangam for over 6 months, have you noticed any changes to the nature of fieldwork or resources available to you in the last 3 months?
- Have you attended the previous focus group?

<u>Health:</u>

- Does this job affect your physical and mental health? How?
- Do you know how to navigate any challenges you have ? (eg. where to eat lunch, access bathrooms)
- Do you have difficulties with accessing bathrooms in the field?
    - Has Amar Seva Sangam provided you with information regarding where and how to access bathrooms?
    - Have you found information provided by Amar Seva Sangam useful?
    - Have you asked parents/families if you can use their bathrooms? Why / Why not?
    - Have you found any other solution to your problem?
- Do you attend work when you are menstruating? What is your experience during this time?
    - How do you feel entering families' homes when you are on your period?
    - Do you plan your schedule for work day so you have a designated public washroom or families' bathroom to change your pad?
    - Has Amar Seva Sangam provided you with any education/ helpful strategies regarding working during menstruation?
    - How has your opinion changed since hearing the presentation on Menstruation: Myths and Facts?
- How do you take care of your health while working as a fieldworker?
- Have you noticed any changes or improvement to health-related concerns in the last few months?
- Have you received any training from Amar Seva Sangam in regards to workers' health?
    - What did you find useful? (Only applicable to CRWs)
    - What did you not find helpful? (CRWs)
    - Was there any topic that was missing from your training? (CRWs)
    - How could the training you received be improved? (CRWs)

<u>Safety:</u>

- Do you have any concerns with regards to safety when you are in the field?
- What strategies do you use to minimize risks when in the field?
- How do you respond in situations where you feel unsafe/ threatened?
    - What resources do you have?
    - How has Amar Seva Sangam advised you to handle these situations?
- Were you provided any training in addressing safety during fieldwork?
    - What has been helpful?
    - Have you implemented any of the strategies?
    - What has not been helpful?
    - How would you improve the safety manual? Do you refer to it when unsure?

<u>Conclusion</u>

- Are there any further comments or experiences you would like to share on safety or health as a female fieldworker?

Focus Group 2 for Service Providers - Child Program Engagement and School Enrollment

Interview Guide for Qualitative Data Collection for Service Providers

*Note : This is a semi-structured interview guide. The interviewer will use these questions as a guide but will follow the lead from the participant(s) as is common in qualitative data collection in order to gain a thorough and comprehensive understanding of participants' thoughts and lived experience. The questions below reflect the nature of the questions that will be asked, however, as an iterative process, questions may be adapted to fit context and conversation during and across interviews.

Preamble:
In this interview, I will ask you about your thoughts and experiences regarding the early intervention program that you have been a part of delivering. Please feel free to be open. We welcome both positive and negative feedback. The information you share will be confidential and summarized with input from others so no one will know who said what, including ASSA management. The information you share will be used to improve service delivery. Results will be presented in reports that may be published, but no information that identifies you will be included in any publication or report. Reports may include some quotes from participants but without names attached and without any details that could allow the reader to know who said what.

Questions will be about a range of topics including (1) school enrollment, and (2) program engagement by families. Do you have any questions for me before we start?

Part 1: School Enrolment

1. From your experiences working with families, what influences whether a child with a disability goes to school or not?
2. Please think of a child that you provide care to that is attending school. Without using her/his name, please tell me about:
    a. What make it possible for that child to be in school?
    b. What was your role in helping that child attend school?
    c. Who else was involved to support the child to attend school, and how?
3. What are the main reasons why children with disabilities drop out of school?
4. What do you think are the differences for girls vs boys?
5. What influences whether a girl child with a disability goes to school?
6. What influences whether a boy child with a disability goes to school?
7. Imagine one year from now and all children with disabilities that you work with are enrolled in school. What has changed to make that happen?

Part 2: Program Engagement / Attendance

1. From your experiences working with families, how do you think they felt about the EI program?
2. What do you think they <u>liked</u> about the early intervention (EI) program?
3. What do you think they <u>did not like</u> about the EI program?
4. In your experience, what facilitated caregivers to participate in the EI program? (probe re. participate in home-based therapy and in center-based therapy)
5. In your experience, what made it difficult or prevented caregivers from participating in the EI program? (probes: time required for visits/therapy home program; distance to travel; cost; home/work obligations; stigma; results seen/benefits expected; personal priorities/values about your child's development; interactions with professionals)

6. For children who did not participate regularly, what do you think would make them more likely to attend the therapy?
7. What can therapists/CRWs/service providers do to improve attendance for therapy?
    a. What could ASSA do to improve attendance for therapy?

Caregiver Interviews - Child Program Engagement and School Enrollment

Interview Guide for Qualitative Data Collection

Preamble:
In this interview, I will ask you about your thoughts and experiences regarding the early intervention program that you are participating in with your child. Please feel free to be open. We welcome both positive and negative feedback. The information you share will be used to improve service delivery. The information you share will be confidential. No one will know who said what, including ASSA management and therapists. Results will be presented in reports that may be published, but no information that identifies you will be included. Reports may include quotes from participants but without names attached and without any details that could allow the reader to know who said what.

Questions will be about a range of topics including: (1) school enrollment, and (2) your thoughts on what helps or is a barrier to you attending therapy appointments. Do you have any questions for me before we begin?

Part 1: School Enrolment

1. Does your child currently attend school?
    a. If yes,
    b. What made it possible for your child to be enrolled in school?
    c. Who is involved in helping your child be enrolled in school? (i.e. be enrolled, stay in school)
    d. What do you and your family do that helps your child attend school?

    e. If no,
    f. Why is your child not currently enrolled in school?
    g. Has your child ever been enrolled in school? If yes, why are they no longer attending school? What would have prevented your child from dropping out of school?
    h. What supports are required to have your child enroll and go to school?
    i. What role do you and your family have to help your child attend school?

2. What do you think influences whether a child with a disability goes to school or not?
3. What do you think are the reasons that children with disabilities drop out of school?
4. What are differences for girls vs boys?
    a. What influences whether a girl child with a disability goes to school?
    b. What influences whether a boy child with a disability goes to school?
5. Imagine one year from now and all children with disabilities are enrolled in school. What has changed to make that happen?

Part 2: Program Engagement

1. What do you like about the early intervention (EI) program?
2. What do you not like about the EI program?
3. What makes it easier for you to participate in the EI program regularly?
4. What makes it more difficult for you to participate in the EI program regularly? (probes: time required for visits/therapy home program; distance to travel; cost; home/work obligations; stigma; results seen/benefits expected; personal priorities/values about your child's development; interactions with professionals)
5. What do you think would make you more likely to attend in future?
    a. What could ASSA do differently?
